# Light-quark and gluon jet discrimination in $pp$ collisions at $\sqrt{s}=7\mathrm {\ TeV}$ with the ATLAS detector

**DOI:** 10.1140/epjc/s10052-014-3023-z

**Published:** 2014-08-21

**Authors:** G. Aad, B. Abbott, J. Abdallah, S. Abdel Khalek, O. Abdinov, R. Aben, B. Abi, M. Abolins, O. S. AbouZeid, H. Abramowicz, H. Abreu, R. Abreu, Y. Abulaiti, B. S. Acharya, L. Adamczyk, D. L. Adams, J. Adelman, S. Adomeit, T. Adye, T. Agatonovic-Jovin, J. A. Aguilar-Saavedra, M. Agustoni, S. P. Ahlen, A. Ahmad, F. Ahmadov, G. Aielli, T. P. A. Åkesson, G. Akimoto, A. V. Akimov, J. Albert, S. Albrand, M. J. Alconada Verzini, M. Aleksa, I. N. Aleksandrov, C. Alexa, G. Alexander, G. Alexandre, T. Alexopoulos, M. Alhroob, G. Alimonti, L. Alio, J. Alison, B. M. M. Allbrooke, L. J. Allison, P. P. Allport, S. E. Allwood-Spiers, J. Almond, A. Aloisio, A. Alonso, F. Alonso, C. Alpigiani, A. Altheimer, B. Alvarez Gonzalez, M. G. Alviggi, K. Amako, Y. Amaral Coutinho, C. Amelung, D. Amidei, S. P. Amor Dos Santos, A. Amorim, S. Amoroso, N. Amram, G. Amundsen, C. Anastopoulos, L. S. Ancu, N. Andari, T. Andeen, C. F. Anders, G. Anders, K. J. Anderson, A. Andreazza, V. Andrei, X. S. Anduaga, S. Angelidakis, I. Angelozzi, P. Anger, A. Angerami, F. Anghinolfi, A. V. Anisenkov, N. Anjos, A. Annovi, A. Antonaki, M. Antonelli, A. Antonov, J. Antos, F. Anulli, M. Aoki, L. Aperio Bella, R. Apolle, G. Arabidze, I. Aracena, Y. Arai, J. P. Araque, A. T. H. Arce, J-F. Arguin, S. Argyropoulos, M. Arik, A. J. Armbruster, O. Arnaez, V. Arnal, H. Arnold, O. Arslan, A. Artamonov, G. Artoni, S. Asai, N. Asbah, A. Ashkenazi, S. Ask, B. Åsman, L. Asquith, K. Assamagan, R. Astalos, M. Atkinson, N. B. Atlay, B. Auerbach, K. Augsten, M. Aurousseau, G. Avolio, G. Azuelos, Y. Azuma, M. A. Baak, C. Bacci, H. Bachacou, K. Bachas, M. Backes, M. Backhaus, J. Backus Mayes, E. Badescu, P. Bagiacchi, P. Bagnaia, Y. Bai, T. Bain, J. T. Baines, O. K. Baker, S. Baker, P. Balek, F. Balli, E. Banas, Sw. Banerjee, D. Banfi, A. Bangert, A. A. E. Bannoura, V. Bansal, H. S. Bansil, L. Barak, S. P. Baranov, E. L. Barberio, D. Barberis, M. Barbero, T. Barillari, M. Barisonzi, T. Barklow, N. Barlow, B. M. Barnett, R. M. Barnett, Z. Barnovska, A. Baroncelli, G. Barone, A. J. Barr, F. Barreiro, J. Barreiro Guimarães da Costa, R. Bartoldus, A. E. Barton, P. Bartos, V. Bartsch, A. Bassalat, A. Basye, R. L. Bates, L. Batkova, J. R. Batley, M. Battistin, F. Bauer, H. S. Bawa, T. Beau, P. H. Beauchemin, R. Beccherle, P. Bechtle, H. P. Beck, K. Becker, S. Becker, M. Beckingham, C. Becot, A. J. Beddall, A. Beddall, S. Bedikian, V. A. Bednyakov, C. P. Bee, L. J. Beemster, T. A. Beermann, M. Begel, K. Behr, C. Belanger-Champagne, P. J. Bell, W. H. Bell, G. Bella, L. Bellagamba, A. Bellerive, M. Bellomo, A. Belloni, K. Belotskiy, O. Beltramello, O. Benary, D. Benchekroun, K. Bendtz, N. Benekos, Y. Benhammou, E. Benhar Noccioli, J. A. Benitez Garcia, D. P. Benjamin, J. R. Bensinger, K. Benslama, S. Bentvelsen, D. Berge, E. Bergeaas Kuutmann, N. Berger, F. Berghaus, E. Berglund, J. Beringer, C. Bernard, P. Bernat, C. Bernius, F. U. Bernlochner, T. Berry, P. Berta, C. Bertella, F. Bertolucci, M. I. Besana, G. J. Besjes, O. Bessidskaia, N. Besson, C. Betancourt, S. Bethke, W. Bhimji, R. M. Bianchi, L. Bianchini, M. Bianco, O. Biebel, S. P. Bieniek, K. Bierwagen, J. Biesiada, M. Biglietti, J. Bilbao De Mendizabal, H. Bilokon, M. Bindi, S. Binet, A. Bingul, C. Bini, C. W. Black, J. E. Black, K. M. Black, D. Blackburn, R. E. Blair, J.-B. Blanchard, T. Blazek, I. Bloch, C. Blocker, W. Blum, U. Blumenschein, G. J. Bobbink, V. S. Bobrovnikov, S. S. Bocchetta, A. Bocci, C. R. Boddy, M. Boehler, J. Boek, T. T. Boek, J. A. Bogaerts, A. G. Bogdanchikov, A. Bogouch, C. Bohm, J. Bohm, V. Boisvert, T. Bold, V. Boldea, A. S. Boldyrev, M. Bomben, M. Bona, M. Boonekamp, A. Borisov, G. Borissov, M. Borri, S. Borroni, J. Bortfeldt, V. Bortolotto, K. Bos, D. Boscherini, M. Bosman, H. Boterenbrood, J. Boudreau, J. Bouffard, E. V. Bouhova-Thacker, D. Boumediene, C. Bourdarios, N. Bousson, S. Boutouil, A. Boveia, J. Boyd, I. R. Boyko, I. Bozovic-Jelisavcic, J. Bracinik, P. Branchini, A. Brandt, G. Brandt, O. Brandt, U. Bratzler, B. Brau, J. E. Brau, H. M. Braun, S. F. Brazzale, B. Brelier, K. Brendlinger, A. J. Brennan, R. Brenner, S. Bressler, K. Bristow, T. M. Bristow, D. Britton, F. M. Brochu, I. Brock, R. Brock, C. Bromberg, J. Bronner, G. Brooijmans, T. Brooks, W. K. Brooks, J. Brosamer, E. Brost, G. Brown, J. Brown, P. A. Bruckman de Renstrom, D. Bruncko, R. Bruneliere, S. Brunet, A. Bruni, G. Bruni, M. Bruschi, L. Bryngemark, T. Buanes, Q. Buat, F. Bucci, P. Buchholz, R. M. Buckingham, A. G. Buckley, S. I. Buda, I. A. Budagov, F. Buehrer, L. Bugge, M. K. Bugge, O. Bulekov, A. C. Bundock, H. Burckhart, S. Burdin, B. Burghgrave, S. Burke, I. Burmeister, E. Busato, D. Büscher, V. Büscher, P. Bussey, C. P. Buszello, B. Butler, J. M. Butler, A. I. Butt, C. M. Buttar, J. M. Butterworth, P. Butti, W. Buttinger, A. Buzatu, M. Byszewski, S. Cabrera Urbán, D. Caforio, O. Cakir, P. Calafiura, A. Calandri, G. Calderini, P. Calfayan, R. Calkins, L. P. Caloba, D. Calvet, S. Calvet, R. Camacho Toro, S. Camarda, D. Cameron, L. M. Caminada, R. Caminal Armadans, S. Campana, M. Campanelli, A. Campoverde, V. Canale, A. Canepa, J. Cantero, R. Cantrill, T. Cao, M. D. M. Capeans Garrido, I. Caprini, M. Caprini, M. Capua, R. Caputo, R. Cardarelli, T. Carli, G. Carlino, L. Carminati, S. Caron, E. Carquin, G. D. Carrillo-Montoya, A. A. Carter, J. R. Carter, J. Carvalho, D. Casadei, M. P. Casado, E. Castaneda-Miranda, A. Castelli, V. Castillo Gimenez, N. F. Castro, P. Catastini, A. Catinaccio, J. R. Catmore, A. Cattai, G. Cattani, S. Caughron, V. Cavaliere, D. Cavalli, M. Cavalli-Sforza, V. Cavasinni, F. Ceradini, B. Cerio, K. Cerny, A. S. Cerqueira, A. Cerri, L. Cerrito, F. Cerutti, M. Cerv, A. Cervelli, S. A. Cetin, A. Chafaq, D. Chakraborty, I. Chalupkova, K. Chan, P. Chang, B. Chapleau, J. D. Chapman, D. Charfeddine, D. G. Charlton, C. C. Chau, C. A. Chavez Barajas, S. Cheatham, A. Chegwidden, S. Chekanov, S. V. Chekulaev, G. A. Chelkov, M. A. Chelstowska, C. Chen, H. Chen, K. Chen, L. Chen, S. Chen, X. Chen, Y. Chen, H. C. Cheng, Y. Cheng, A. Cheplakov, R. Cherkaoui El Moursli, V. Chernyatin, E. Cheu, L. Chevalier, V. Chiarella, G. Chiefari, J. T. Childers, A. Chilingarov, G. Chiodini, A. S. Chisholm, R. T. Chislett, A. Chitan, M. V. Chizhov, S. Chouridou, B. K. B. Chow, I. A. Christidi, D. Chromek-Burckhart, M. L. Chu, J. Chudoba, J. J. Chwastowski, L. Chytka, G. Ciapetti, A. K. Ciftci, R. Ciftci, D. Cinca, V. Cindro, A. Ciocio, P. Cirkovic, Z. H. Citron, M. Citterio, M. Ciubancan, A. Clark, P. J. Clark, R. N. Clarke, W. Cleland, J. C. Clemens, C. Clement, Y. Coadou, M. Cobal, A. Coccaro, J. Cochran, L. Coffey, J. G. Cogan, J. Coggeshall, B. Cole, S. Cole, A. P. Colijn, C. Collins-Tooth, J. Collot, T. Colombo, G. Colon, G. Compostella, P. Conde Muiño, E. Coniavitis, M. C. Conidi, S. H. Connell, I. A. Connelly, S. M. Consonni, V. Consorti, S. Constantinescu, C. Conta, G. Conti, F. Conventi, M. Cooke, B. D. Cooper, A. M. Cooper-Sarkar, N. J. Cooper-Smith, K. Copic, T. Cornelissen, M. Corradi, F. Corriveau, A. Corso-Radu, A. Cortes-Gonzalez, G. Cortiana, G. Costa, M. J. Costa, D. Costanzo, D. Côté, G. Cottin, G. Cowan, B. E. Cox, K. Cranmer, G. Cree, S. Crépé-Renaudin, F. Crescioli, M. Crispin Ortuzar, M. Cristinziani, V. Croft, G. Crosetti, C.-M. Cuciuc, C. Cuenca Almenar, T. Cuhadar Donszelmann, J. Cummings, M. Curatolo, C. Cuthbert, H. Czirr, P. Czodrowski, Z. Czyczula, S. D’Auria, M. D’Onofrio, M. J. Da Cunha Sargedas De Sousa, C. Da Via, W. Dabrowski, A. Dafinca, T. Dai, O. Dale, F. Dallaire, C. Dallapiccola, M. Dam, A. C. Daniells, M. Dano Hoffmann, V. Dao, G. Darbo, G. L. Darlea, S. Darmora, J. A. Dassoulas, A. Dattagupta, W. Davey, C. David, T. Davidek, E. Davies, M. Davies, O. Davignon, A. R. Davison, P. Davison, Y. Davygora, E. Dawe, I. Dawson, R. K. Daya-Ishmukhametova, K. De, R. de Asmundis, S. De Castro, S. De Cecco, J. de Graat, N. De Groot, P. de Jong, H. De la Torre, F. De Lorenzi, L. De Nooij, D. De Pedis, A. De Salvo, U. De Sanctis, A. De Santo, J. B. De Vivie De Regie, G. De Zorzi, W. J. Dearnaley, R. Debbe, C. Debenedetti, B. Dechenaux, D. V. Dedovich, J. Degenhardt, I. Deigaard, J. Del Peso, T. Del Prete, F. Deliot, C. M. Delitzsch, M. Deliyergiyev, A. Dell’Acqua, L. Dell’Asta, M. Dell’Orso, M. Della Pietra, D. della Volpe, M. Delmastro, P. A. Delsart, C. Deluca, S. Demers, M. Demichev, A. Demilly, S. P. Denisov, D. Derendarz, J. E. Derkaoui, F. Derue, P. Dervan, K. Desch, C. Deterre, P. O. Deviveiros, A. Dewhurst, S. Dhaliwal, A. Di Ciaccio, L. Di Ciaccio, A. Di Domenico, C. Di Donato, A. Di Girolamo, B. Di Girolamo, A. Di Mattia, B. Di Micco, R. Di Nardo, A. Di Simone, R. Di Sipio, D. Di Valentino, M. A. Diaz, E. B. Diehl, J. Dietrich, T. A. Dietzsch, S. Diglio, A. Dimitrievska, J. Dingfelder, C. Dionisi, P. Dita, S. Dita, F. Dittus, F. Djama, T. Djobava, M. A. B. do Vale, A. Do Valle Wemans, T. K. O. Doan, D. Dobos, E. Dobson, C. Doglioni, T. Doherty, T. Dohmae, J. Dolejsi, Z. Dolezal, B. A. Dolgoshein, M. Donadelli, S. Donati, P. Dondero, J. Donini, J. Dopke, A. Doria, A. Dos Anjos, M. T. Dova, A. T. Doyle, M. Dris, J. Dubbert, S. Dube, E. Dubreuil, E. Duchovni, G. Duckeck, O. A. Ducu, D. Duda, A. Dudarev, F. Dudziak, L. Duflot, L. Duguid, M. Dührssen, M. Dunford, H. Duran Yildiz, M. Düren, A. Durglishvili, M. Dwuznik, M. Dyndal, J. Ebke, W. Edson, N. C. Edwards, W. Ehrenfeld, T. Eifert, G. Eigen, K. Einsweiler, T. Ekelof, M. El Kacimi, M. Ellert, S. Elles, F. Ellinghaus, N. Ellis, J. Elmsheuser, M. Elsing, D. Emeliyanov, Y. Enari, O. C. Endner, M. Endo, R. Engelmann, J. Erdmann, A. Ereditato, D. Eriksson, G. Ernis, J. Ernst, M. Ernst, J. Ernwein, D. Errede, S. Errede, E. Ertel, M. Escalier, H. Esch, C. Escobar, B. Esposito, A. I. Etienvre, E. Etzion, H. Evans, L. Fabbri, G. Facini, R. M. Fakhrutdinov, S. Falciano, J. Faltova, Y. Fang, M. Fanti, A. Farbin, A. Farilla, T. Farooque, S. Farrell, S. M. Farrington, P. Farthouat, F. Fassi, P. Fassnacht, D. Fassouliotis, A. Favareto, L. Fayard, P. Federic, O. L. Fedin, W. Fedorko, M. Fehling-Kaschek, S. Feigl, L. Feligioni, C. Feng, E. J. Feng, H. Feng, A. B. Fenyuk, S. Fernandez Perez, S. Ferrag, J. Ferrando, A. Ferrari, P. Ferrari, R. Ferrari, D. E. Ferreira de Lima, A. Ferrer, D. Ferrere, C. Ferretti, A. Ferretto Parodi, M. Fiascaris, F. Fiedler, A. Filipčič, M. Filipuzzi, F. Filthaut, M. Fincke-Keeler, K. D. Finelli, M. C. N. Fiolhais, L. Fiorini, A. Firan, J. Fischer, W. C. Fisher, E. A. Fitzgerald, M. Flechl, I. Fleck, P. Fleischmann, S. Fleischmann, G. T. Fletcher, G. Fletcher, T. Flick, A. Floderus, L. R. Flores Castillo, A. C. Florez Bustos, M. J. Flowerdew, A. Formica, A. Forti, D. Fortin, D. Fournier, H. Fox, S. Fracchia, P. Francavilla, M. Franchini, S. Franchino, D. Francis, M. Franklin, S. Franz, M. Fraternali, S. T. French, C. Friedrich, F. Friedrich, D. Froidevaux, J. A. Frost, C. Fukunaga, E. Fullana Torregrosa, B. G. Fulsom, J. Fuster, C. Gabaldon, O. Gabizon, A. Gabrielli, A. Gabrielli, S. Gadatsch, S. Gadomski, G. Gagliardi, P. Gagnon, C. Galea, B. Galhardo, E. J. Gallas, V. Gallo, B. J. Gallop, P. Gallus, G. Galster, K. K. Gan, R. P. Gandrajula, J. Gao, Y. S. Gao, F. M. Garay Walls, F. Garberson, C. García, J. E. García Navarro, M. Garcia-Sciveres, R. W. Gardner, N. Garelli, V. Garonne, C. Gatti, G. Gaudio, B. Gaur, L. Gauthier, P. Gauzzi, I. L. Gavrilenko, C. Gay, G. Gaycken, E. N. Gazis, P. Ge, Z. Gecse, C. N. P. Gee, D. A. A. Geerts, Ch. Geich-Gimbel, K. Gellerstedt, C. Gemme, A. Gemmell, M. H. Genest, S. Gentile, M. George, S. George, D. Gerbaudo, A. Gershon, H. Ghazlane, N. Ghodbane, B. Giacobbe, S. Giagu, V. Giangiobbe, P. Giannetti, F. Gianotti, B. Gibbard, S. M. Gibson, M. Gilchriese, T. P. S. Gillam, D. Gillberg, G. Gilles, D. M. Gingrich, N. Giokaris, M. P. Giordani, R. Giordano, F. M. Giorgi, P. F. Giraud, D. Giugni, C. Giuliani, M. Giulini, B. K. Gjelsten, I. Gkialas, L. K. Gladilin, C. Glasman, J. Glatzer, P. C. F. Glaysher, A. Glazov, G. L. Glonti, M. Goblirsch-Kolb, J. R. Goddard, J. Godfrey, J. Godlewski, C. Goeringer, S. Goldfarb, T. Golling, D. Golubkov, A. Gomes, L. S. Gomez Fajardo, R. Gonçalo, J. Goncalves Pinto Firmino Da Costa, L. Gonella, S. González de la Hoz, G. Gonzalez Parra, M. L. Gonzalez Silva, S. Gonzalez-Sevilla, L. Goossens, P. A. Gorbounov, H. A. Gordon, I. Gorelov, G. Gorfine, B. Gorini, E. Gorini, A. Gorišek, E. Gornicki, A. T. Goshaw, C. Gössling, M. I. Gostkin, M. Gouighri, D. Goujdami, M. P. Goulette, A. G. Goussiou, C. Goy, S. Gozpinar, H. M. X. Grabas, L. Graber, I. Grabowska-Bold, P. Grafström, K-J. Grahn, J. Gramling, E. Gramstad, S. Grancagnolo, V. Grassi, V. Gratchev, H. M. Gray, E. Graziani, O. G. Grebenyuk, Z. D. Greenwood, K. Gregersen, I. M. Gregor, P. Grenier, J. Griffiths, N. Grigalashvili, A. A. Grillo, K. Grimm, S. Grinstein, Ph. Gris, Y. V. Grishkevich, J.-F. Grivaz, J. P. Grohs, A. Grohsjean, E. Gross, J. Grosse-Knetter, G. C. Grossi, J. Groth-Jensen, Z. J. Grout, K. Grybel, L. Guan, F. Guescini, D. Guest, O. Gueta, C. Guicheney, E. Guido, T. Guillemin, S. Guindon, U. Gul, C. Gumpert, J. Gunther, J. Guo, S. Gupta, P. Gutierrez, N. G. Gutierrez Ortiz, C. Gutschow, N. Guttman, C. Guyot, C. Gwenlan, C. B. Gwilliam, A. Haas, C. Haber, H. K. Hadavand, N. Haddad, P. Haefner, S. Hageboeck, Z. Hajduk, H. Hakobyan, M. Haleem, D. Hall, G. Halladjian, K. Hamacher, P. Hamal, K. Hamano, M. Hamer, A. Hamilton, S. Hamilton, P. G. Hamnett, L. Han, K. Hanagaki, K. Hanawa, M. Hance, P. Hanke, J. B. Hansen, J. D. Hansen, P. H. Hansen, K. Hara, A. S. Hard, T. Harenberg, S. Harkusha, D. Harper, R. D. Harrington, O. M. Harris, P. F. Harrison, F. Hartjes, S. Hasegawa, Y. Hasegawa, A. Hasib, S. Hassani, S. Haug, M. Hauschild, R. Hauser, M. Havranek, C. M. Hawkes, R. J. Hawkings, A. D. Hawkins, T. Hayashi, D. Hayden, C. P. Hays, H. S. Hayward, S. J. Haywood, S. J. Head, T. Heck, V. Hedberg, L. Heelan, S. Heim, T. Heim, B. Heinemann, L. Heinrich, S. Heisterkamp, J. Hejbal, L. Helary, C. Heller, M. Heller, S. Hellman, D. Hellmich, C. Helsens, J. Henderson, R. C. W. Henderson, C. Hengler, A. Henrichs, A. M. Henriques Correia, S. Henrot-Versille, C. Hensel, G. H. Herbert, Y. Hernández Jiménez, R. Herrberg-Schubert, G. Herten, R. Hertenberger, L. Hervas, G. G. Hesketh, N. P. Hessey, R. Hickling, E. Higón-Rodriguez, J. C. Hill, K. H. Hiller, S. Hillert, S. J. Hillier, I. Hinchliffe, E. Hines, M. Hirose, D. Hirschbuehl, J. Hobbs, N. Hod, M. C. Hodgkinson, P. Hodgson, A. Hoecker, M. R. Hoeferkamp, J. Hoffman, D. Hoffmann, J. I. Hofmann, M. Hohlfeld, T. R. Holmes, T. M. Hong, L. Hooft van Huysduynen, J-Y. Hostachy, S. Hou, A. Hoummada, J. Howard, J. Howarth, M. Hrabovsky, I. Hristova, J. Hrivnac, T. Hryn’ova, P. J. Hsu, S.-C. Hsu, D. Hu, X. Hu, Y. Huang, Z. Hubacek, F. Hubaut, F. Huegging, T. B. Huffman, E. W. Hughes, G. Hughes, M. Huhtinen, T. A. Hülsing, M. Hurwitz, N. Huseynov, J. Huston, J. Huth, G. Iacobucci, G. Iakovidis, I. Ibragimov, L. Iconomidou-Fayard, J. Idarraga, E. Ideal, P. Iengo, O. Igonkina, T. Iizawa, Y. Ikegami, K. Ikematsu, M. Ikeno, D. Iliadis, N. Ilic, Y. Inamaru, T. Ince, P. Ioannou, M. Iodice, K. Iordanidou, V. Ippolito, A. Irles Quiles, C. Isaksson, M. Ishino, M. Ishitsuka, R. Ishmukhametov, C. Issever, S. Istin, J. M. Iturbe Ponce, J. Ivarsson, A. V. Ivashin, W. Iwanski, H. Iwasaki, J. M. Izen, V. Izzo, B. Jackson, J. N. Jackson, M. Jackson, P. Jackson, M. R. Jaekel, V. Jain, K. Jakobs, S. Jakobsen, T. Jakoubek, J. Jakubek, D. O. Jamin, D. K. Jana, E. Jansen, H. Jansen, J. Janssen, M. Janus, G. Jarlskog, N. Javadov, T. Javůrek, L. Jeanty, G.-Y. Jeng, D. Jennens, P. Jenni, J. Jentzsch, C. Jeske, S. Jézéquel, H. Ji, W. Ji, J. Jia, Y. Jiang, M. Jimenez Belenguer, S. Jin, A. Jinaru, O. Jinnouchi, M. D. Joergensen, K. E. Johansson, P. Johansson, K. A. Johns, K. Jon-And, G. Jones, R. W. L. Jones, T. J. Jones, J. Jongmanns, P. M. Jorge, K. D. Joshi, J. Jovicevic, X. Ju, C. A. Jung, R. M. Jungst, P. Jussel, A. Juste Rozas, M. Kaci, A. Kaczmarska, M. Kado, H. Kagan, M. Kagan, E. Kajomovitz, S. Kama, N. Kanaya, M. Kaneda, S. Kaneti, T. Kanno, V. A. Kantserov, J. Kanzaki, B. Kaplan, A. Kapliy, D. Kar, K. Karakostas, N. Karastathis, M. Karnevskiy, S. N. Karpov, K. Karthik, V. Kartvelishvili, A. N. Karyukhin, L. Kashif, G. Kasieczka, R. D. Kass, A. Kastanas, Y. Kataoka, A. Katre, J. Katzy, V. Kaushik, K. Kawagoe, T. Kawamoto, G. Kawamura, S. Kazama, V. F. Kazanin, M. Y. Kazarinov, R. Keeler, P. T. Keener, R. Kehoe, M. Keil, J. S. Keller, H. Keoshkerian, O. Kepka, B. P. Kerševan, S. Kersten, K. Kessoku, J. Keung, F. Khalil-zada, H. Khandanyan, A. Khanov, A. Khodinov, A. Khomich, T. J. Khoo, G. Khoriauli, A. Khoroshilov, V. Khovanskiy, E. Khramov, J. Khubua, H. Y. Kim, H. Kim, S. H. Kim, N. Kimura, O. Kind, B. T. King, M. King, R. S. B. King, S. B. King, J. Kirk, A. E. Kiryunin, T. Kishimoto, D. Kisielewska, F. Kiss, T. Kitamura, T. Kittelmann, K. Kiuchi, E. Kladiva, M. Klein, U. Klein, K. Kleinknecht, P. Klimek, A. Klimentov, R. Klingenberg, J. A. Klinger, T. Klioutchnikova, P. F. Klok, E.-E. Kluge, P. Kluit, S. Kluth, E. Kneringer, E. B. F. G. Knoops, A. Knue, T. Kobayashi, M. Kobel, M. Kocian, P. Kodys, P. Koevesarki, T. Koffas, E. Koffeman, L. A. Kogan, S. Kohlmann, Z. Kohout, T. Kohriki, T. Koi, H. Kolanoski, I. Koletsou, J. Koll, A. A. Komar, Y. Komori, T. Kondo, N. Kondrashova, K. Köneke, A. C. König, S. König, T. Kono, R. Konoplich, N. Konstantinidis, R. Kopeliansky, S. Koperny, L. Köpke, A. K. Kopp, K. Korcyl, K. Kordas, A. Korn, A. A. Korol, I. Korolkov, E. V. Korolkova, V. A. Korotkov, O. Kortner, S. Kortner, V. V. Kostyukhin, S. Kotov, V. M. Kotov, A. Kotwal, C. Kourkoumelis, V. Kouskoura, A. Koutsman, R. Kowalewski, T. Z. Kowalski, W. Kozanecki, A. S. Kozhin, V. Kral, V. A. Kramarenko, G. Kramberger, D. Krasnopevtsev, M. W. Krasny, A. Krasznahorkay, J. K. Kraus, A. Kravchenko, S. Kreiss, M. Kretz, J. Kretzschmar, K. Kreutzfeldt, P. Krieger, K. Kroeninger, H. Kroha, J. Kroll, J. Kroseberg, J. Krstic, U. Kruchonak, H. Krüger, T. Kruker, N. Krumnack, Z. V. Krumshteyn, A. Kruse, M. C. Kruse, M. Kruskal, T. Kubota, S. Kuday, S. Kuehn, A. Kugel, A. Kuhl, T. Kuhl, V. Kukhtin, Y. Kulchitsky, S. Kuleshov, M. Kuna, J. Kunkle, A. Kupco, H. Kurashige, Y. A. Kurochkin, R. Kurumida, V. Kus, E. S. Kuwertz, M. Kuze, J. Kvita, A. La Rosa, L. La Rotonda, C. Lacasta, F. Lacava, J. Lacey, H. Lacker, D. Lacour, V. R. Lacuesta, E. Ladygin, R. Lafaye, B. Laforge, T. Lagouri, S. Lai, H. Laier, L. Lambourne, S. Lammers, C. L. Lampen, W. Lampl, E. Lançon, U. Landgraf, M. P. J. Landon, V. S. Lang, C. Lange, A. J. Lankford, F. Lanni, K. Lantzsch, S. Laplace, C. Lapoire, J. F. Laporte, T. Lari, M. Lassnig, P. Laurelli, W. Lavrijsen, A. T. Law, P. Laycock, B. T. Le, O. Le Dortz, E. Le Guirriec, E. Le Menedeu, T. LeCompte, F. Ledroit-Guillon, C. A. Lee, H. Lee, J. S. H. Lee, S. C. Lee, L. Lee, G. Lefebvre, M. Lefebvre, F. Legger, C. Leggett, A. Lehan, M. Lehmacher, G. Lehmann Miotto, X. Lei, A. G. Leister, M. A. L. Leite, R. Leitner, D. Lellouch, B. Lemmer, K. J. C. Leney, T. Lenz, G. Lenzen, B. Lenzi, R. Leone, K. Leonhardt, S. Leontsinis, C. Leroy, C. G. Lester, C. M. Lester, M. Levchenko, J. Levêque, D. Levin, L. J. Levinson, M. Levy, A. Lewis, G. H. Lewis, A. M. Leyko, M. Leyton, B. Li, B. Li, H. Li, H. L. Li, L. Li, S. Li, Y. Li, Z. Liang, H. Liao, B. Liberti, P. Lichard, K. Lie, J. Liebal, W. Liebig, C. Limbach, A. Limosani, M. Limper, S. C. Lin, F. Linde, B. E. Lindquist, J. T. Linnemann, E. Lipeles, A. Lipniacka, M. Lisovyi, T. M. Liss, D. Lissauer, A. Lister, A. M. Litke, B. Liu, D. Liu, J. B. Liu, K. Liu, L. Liu, M. Liu, M. Liu, Y. Liu, M. Livan, S. S. A. Livermore, A. Lleres, J. Llorente Merino, S. L. Lloyd, F. Lo Sterzo, E. Lobodzinska, P. Loch, W. S. Lockman, T. Loddenkoetter, F. K. Loebinger, A. E. Loevschall-Jensen, A. Loginov, C. W. Loh, T. Lohse, K. Lohwasser, M. Lokajicek, V. P. Lombardo, B. A. Long, J. D. Long, R. E. Long, L. Lopes, D. Lopez Mateos, B. Lopez Paredes, J. Lorenz, N. Lorenzo Martinez, M. Losada, P. Loscutoff, X. Lou, A. Lounis, J. Love, P. A. Love, A. J. Lowe, F. Lu, H. J. Lubatti, C. Luci, A. Lucotte, F. Luehring, W. Lukas, L. Luminari, O. Lundberg, B. Lund-Jensen, M. Lungwitz, D. Lynn, R. Lysak, E. Lytken, H. Ma, L. L. Ma, G. Maccarrone, A. Macchiolo, J. Machado Miguens, D. Macina, D. Madaffari, R. Madar, H. J. Maddocks, W. F. Mader, A. Madsen, M. Maeno, T. Maeno, E. Magradze, K. Mahboubi, J. Mahlstedt, S. Mahmoud, C. Maiani, C. Maidantchik, A. Maio, S. Majewski, Y. Makida, N. Makovec, P. Mal, B. Malaescu, Pa. Malecki, V. P. Maleev, F. Malek, U. Mallik, D. Malon, C. Malone, S. Maltezos, V. M. Malyshev, S. Malyukov, J. Mamuzic, B. Mandelli, L. Mandelli, I. Mandić, R. Mandrysch, J. Maneira, A. Manfredini, L. Manhaes de Andrade Filho, J. A. Manjarres Ramos, A. Mann, P. M. Manning, A. Manousakis-Katsikakis, B. Mansoulie, R. Mantifel, L. Mapelli, L. March, J. F. Marchand, G. Marchiori, M. Marcisovsky, C. P. Marino, C. N. Marques, F. Marroquim, S. P. Marsden, Z. Marshall, L. F. Marti, S. Marti-Garcia, B. Martin, B. Martin, J. P. Martin, T. A. Martin, V. J. Martin, B. Martin dit Latour, H. Martinez, M. Martinez, S. Martin-Haugh, A. C. Martyniuk, M. Marx, F. Marzano, A. Marzin, L. Masetti, T. Mashimo, R. Mashinistov, J. Masik, A. L. Maslennikov, I. Massa, N. Massol, P. Mastrandrea, A. Mastroberardino, T. Masubuchi, P. Matricon, H. Matsunaga, T. Matsushita, P. Mättig, S. Mättig, J. Mattmann, J. Maurer, S. J. Maxfield, D. A. Maximov, R. Mazini, L. Mazzaferro, G. Mc Goldrick, S. P. Mc Kee, A. McCarn, R. L. McCarthy, T. G. McCarthy, N. A. McCubbin, K. W. McFarlane, J. A. Mcfayden, G. Mchedlidze, T. Mclaughlan, S. J. McMahon, R. A. McPherson, A. Meade, J. Mechnich, M. Medinnis, S. Meehan, S. Mehlhase, A. Mehta, K. Meier, C. Meineck, B. Meirose, C. Melachrinos, B. R. Mellado Garcia, F. Meloni, A. Mengarelli, S. Menke, E. Meoni, K. M. Mercurio, S. Mergelmeyer, N. Meric, P. Mermod, L. Merola, C. Meroni, F. S. Merritt, H. Merritt, A. Messina, J. Metcalfe, A. S. Mete, C. Meyer, C. Meyer, J-P. Meyer, J. Meyer, R. P. Middleton, S. Migas, L. Mijović, G. Mikenberg, M. Mikestikova, M. Mikuž, D. W. Miller, C. Mills, A. Milov, D. A. Milstead, D. Milstein, A. A. Minaenko, M. Miñano Moya, I. A. Minashvili, A. I. Mincer, B. Mindur, M. Mineev, Y. Ming, L. M. Mir, G. Mirabelli, T. Mitani, J. Mitrevski, V. A. Mitsou, S. Mitsui, A. Miucci, P. S. Miyagawa, J. U. Mjörnmark, T. Moa, K. Mochizuki, V. Moeller, S. Mohapatra, W. Mohr, S. Molander, R. Moles-Valls, K. Mönig, C. Monini, J. Monk, E. Monnier, J. Montejo Berlingen, F. Monticelli, S. Monzani, R. W. Moore, A. Moraes, N. Morange, J. Morel, D. Moreno, M. Moreno Llácer, P. Morettini, M. Morgenstern, M. Morii, S. Moritz, A. K. Morley, G. Mornacchi, J. D. Morris, L. Morvaj, H. G. Moser, M. Mosidze, J. Moss, R. Mount, E. Mountricha, S. V. Mouraviev, E. J. W. Moyse, S. Muanza, R. D. Mudd, F. Mueller, J. Mueller, K. Mueller, T. Mueller, T. Mueller, D. Muenstermann, Y. Munwes, J. A. Murillo Quijada, W. J. Murray, H. Musheghyan, E. Musto, A. G. Myagkov, M. Myska, O. Nackenhorst, J. Nadal, K. Nagai, R. Nagai, Y. Nagai, K. Nagano, A. Nagarkar, Y. Nagasaka, M. Nagel, A. M. Nairz, Y. Nakahama, K. Nakamura, T. Nakamura, I. Nakano, H. Namasivayam, G. Nanava, R. Narayan, T. Nattermann, T. Naumann, G. Navarro, R. Nayyar, H. A. Neal, P. Yu. Nechaeva, T. J. Neep, A. Negri, G. Negri, M. Negrini, S. Nektarijevic, A. Nelson, T. K. Nelson, S. Nemecek, P. Nemethy, A. A. Nepomuceno, M. Nessi, M. S. Neubauer, M. Neumann, R. M. Neves, P. Nevski, F. M. Newcomer, P. R. Newman, D. H. Nguyen, R. B. Nickerson, R. Nicolaidou, B. Nicquevert, J. Nielsen, N. Nikiforou, A. Nikiforov, V. Nikolaenko, I. Nikolic-Audit, K. Nikolics, K. Nikolopoulos, P. Nilsson, Y. Ninomiya, A. Nisati, R. Nisius, T. Nobe, L. Nodulman, M. Nomachi, I. Nomidis, S. Norberg, M. Nordberg, S. Nowak, M. Nozaki, L. Nozka, K. Ntekas, G. Nunes Hanninger, T. Nunnemann, E. Nurse, F. Nuti, B. J. O’Brien, F. O’grady, D. C. O’Neil, V. O’Shea, F. G. Oakham, H. Oberlack, T. Obermann, J. Ocariz, A. Ochi, M. I. Ochoa, S. Oda, S. Odaka, H. Ogren, A. Oh, S. H. Oh, C. C. Ohm, H. Ohman, T. Ohshima, W. Okamura, H. Okawa, Y. Okumura, T. Okuyama, A. Olariu, A. G. Olchevski, S. A. Olivares Pino, D. Oliveira Damazio, E. Oliver Garcia, A. Olszewski, J. Olszowska, A. Onofre, P. U. E. Onyisi, C. J. Oram, M. J. Oreglia, Y. Oren, D. Orestano, N. Orlando, C. Oropeza Barrera, R. S. Orr, B. Osculati, R. Ospanov, G. Otero y Garzon, H. Otono, M. Ouchrif, E. A. Ouellette, F. Ould-Saada, A. Ouraou, K. P. Oussoren, Q. Ouyang, A. Ovcharova, M. Owen, V. E. Ozcan, N. Ozturk, K. Pachal, A. Pacheco Pages, C. Padilla Aranda, M. Pagáčová, S. Pagan Griso, E. Paganis, C. Pahl, F. Paige, P. Pais, K. Pajchel, G. Palacino, S. Palestini, D. Pallin, A. Palma, J. D. Palmer, Y. B. Pan, E. Panagiotopoulou, J. G. Panduro Vazquez, P. Pani, N. Panikashvili, S. Panitkin, D. Pantea, L. Paolozzi, Th. D. Papadopoulou, K. Papageorgiou, A. Paramonov, D. Paredes Hernandez, M. A. Parker, F. Parodi, J. A. Parsons, U. Parzefall, E. Pasqualucci, S. Passaggio, A. Passeri, F. Pastore, Fr. Pastore, G. Pásztor, S. Pataraia, N. D. Patel, J. R. Pater, S. Patricelli, T. Pauly, J. Pearce, M. Pedersen, S. Pedraza Lopez, R. Pedro, S. V. Peleganchuk, D. Pelikan, H. Peng, B. Penning, J. Penwell, D. V. Perepelitsa, E. Perez Codina, M. T. Pérez García-Estañ, V. Perez Reale, L. Perini, H. Pernegger, R. Perrino, R. Peschke, V. D. Peshekhonov, K. Peters, R. F. Y. Peters, B. A. Petersen, J. Petersen, T. C. Petersen, E. Petit, A. Petridis, C. Petridou, E. Petrolo, F. Petrucci, M. Petteni, N. E. Pettersson, R. Pezoa, P. W. Phillips, G. Piacquadio, E. Pianori, A. Picazio, E. Piccaro, M. Piccinini, R. Piegaia, D. T. Pignotti, J. E. Pilcher, A. D. Pilkington, J. Pina, M. Pinamonti, A. Pinder, J. L. Pinfold, A. Pingel, B. Pinto, S. Pires, M. Pitt, C. Pizio, M.-A. Pleier, V. Pleskot, E. Plotnikova, P. Plucinski, S. Poddar, F. Podlyski, R. Poettgen, L. Poggioli, D. Pohl, M. Pohl, G. Polesello, A. Policicchio, R. Polifka, A. Polini, C. S. Pollard, V. Polychronakos, K. Pommès, L. Pontecorvo, B. G. Pope, G. A. Popeneciu, D. S. Popovic, A. Poppleton, X. Portell Bueso, G. E. Pospelov, S. Pospisil, K. Potamianos, I. N. Potrap, C. J. Potter, C. T. Potter, G. Poulard, J. Poveda, V. Pozdnyakov, P. Pralavorio, A. Pranko, S. Prasad, R. Pravahan, S. Prell, D. Price, J. Price, L. E. Price, D. Prieur, M. Primavera, M. Proissl, K. Prokofiev, F. Prokoshin, E. Protopapadaki, S. Protopopescu, J. Proudfoot, M. Przybycien, H. Przysiezniak, E. Ptacek, E. Pueschel, D. Puldon, M. Purohit, P. Puzo, J. Qian, G. Qin, Y. Qin, A. Quadt, D. R. Quarrie, W. B. Quayle, D. Quilty, A. Qureshi, V. Radeka, V. Radescu, S. K. Radhakrishnan, P. Radloff, P. Rados, F. Ragusa, G. Rahal, S. Rajagopalan, M. Rammensee, A. S. Randle-Conde, C. Rangel-Smith, K. Rao, F. Rauscher, T. C. Rave, T. Ravenscroft, M. Raymond, A. L. Read, D. M. Rebuzzi, A. Redelbach, G. Redlinger, R. Reece, K. Reeves, L. Rehnisch, A. Reinsch, H. Reisin, M. Relich, C. Rembser, Z. L. Ren, A. Renaud, M. Rescigno, S. Resconi, B. Resende, O. L. Rezanova, P. Reznicek, R. Rezvani, R. Richter, M. Ridel, P. Rieck, M. Rijssenbeek, A. Rimoldi, L. Rinaldi, E. Ritsch, I. Riu, F. Rizatdinova, E. Rizvi, S. H. Robertson, A. Robichaud-Veronneau, D. Robinson, J. E. M. Robinson, A. Robson, C. Roda, L. Rodrigues, S. Roe, O. Røhne, S. Rolli, A. Romaniouk, M. Romano, G. Romeo, E. Romero Adam, N. Rompotis, L. Roos, E. Ros, S. Rosati, K. Rosbach, M. Rose, P. L. Rosendahl, O. Rosenthal, V. Rossetti, E. Rossi, L. P. Rossi, R. Rosten, M. Rotaru, I. Roth, J. Rothberg, D. Rousseau, C. R. Royon, A. Rozanov, Y. Rozen, X. Ruan, F. Rubbo, I. Rubinskiy, V. I. Rud, C. Rudolph, M. S. Rudolph, F. Rühr, A. Ruiz-Martinez, Z. Rurikova, N. A. Rusakovich, A. Ruschke, J. P. Rutherfoord, N. Ruthmann, Y. F. Ryabov, M. Rybar, G. Rybkin, N. C. Ryder, A. F. Saavedra, S. Sacerdoti, A. Saddique, I. Sadeh, H. F-W. Sadrozinski, R. Sadykov, F. Safai Tehrani, H. Sakamoto, Y. Sakurai, G. Salamanna, A. Salamon, M. Saleem, D. Salek, P. H. Sales De Bruin, D. Salihagic, A. Salnikov, J. Salt, B. M. Salvachua Ferrando, D. Salvatore, F. Salvatore, A. Salvucci, A. Salzburger, D. Sampsonidis, A. Sanchez, J. Sánchez, V. Sanchez Martinez, H. Sandaker, R. L. Sandbach, H. G. Sander, M. P. Sanders, M. Sandhoff, T. Sandoval, C. Sandoval, R. Sandstroem, D. P. C. Sankey, A. Sansoni, C. Santoni, R. Santonico, H. Santos, I. Santoyo Castillo, K. Sapp, A. Sapronov, J. G. Saraiva, B. Sarrazin, G. Sartisohn, O. Sasaki, Y. Sasaki, I. Satsounkevitch, G. Sauvage, E. Sauvan, P. Savard, D. O. Savu, C. Sawyer, L. Sawyer, J. Saxon, C. Sbarra, A. Sbrizzi, T. Scanlon, D. A. Scannicchio, M. Scarcella, J. Schaarschmidt, P. Schacht, D. Schaefer, R. Schaefer, S. Schaepe, S. Schaetzel, U. Schäfer, A. C. Schaffer, D. Schaile, R. D. Schamberger, V. Scharf, V. A. Schegelsky, D. Scheirich, M. Schernau, M. I. Scherzer, C. Schiavi, J. Schieck, C. Schillo, M. Schioppa, S. Schlenker, E. Schmidt, K. Schmieden, C. Schmitt, C. Schmitt, S. Schmitt, B. Schneider, Y. J. Schnellbach, U. Schnoor, L. Schoeffel, A. Schoening, B. D. Schoenrock, A. L. S. Schorlemmer, M. Schott, D. Schouten, J. Schovancova, M. Schram, S. Schramm, M. Schreyer, C. Schroeder, N. Schuh, M. J. Schultens, H.-C. Schultz-Coulon, H. Schulz, M. Schumacher, B. A. Schumm, Ph. Schune, A. Schwartzman, Ph. Schwegler, Ph. Schwemling, R. Schwienhorst, J. Schwindling, T. Schwindt, M. Schwoerer, F. G. Sciacca, E. Scifo, G. Sciolla, W. G. Scott, F. Scuri, F. Scutti, J. Searcy, G. Sedov, E. Sedykh, S. C. Seidel, A. Seiden, F. Seifert, J. M. Seixas, G. Sekhniaidze, S. J. Sekula, K. E. Selbach, D. M. Seliverstov, G. Sellers, N. Semprini-Cesari, C. Serfon, L. Serin, L. Serkin, T. Serre, R. Seuster, H. Severini, F. Sforza, A. Sfyrla, E. Shabalina, M. Shamim, L. Y. Shan, J. T. Shank, Q. T. Shao, M. Shapiro, P. B. Shatalov, K. Shaw, P. Sherwood, S. Shimizu, C. O. Shimmin, M. Shimojima, M. Shiyakova, A. Shmeleva, M. J. Shochet, D. Short, S. Shrestha, E. Shulga, M. A. Shupe, S. Shushkevich, P. Sicho, D. Sidorov, A. Sidoti, F. Siegert, Dj. Sijacki, O. Silbert, J. Silva, Y. Silver, D. Silverstein, S. B. Silverstein, V. Simak, O. Simard, Lj. Simic, S. Simion, E. Simioni, B. Simmons, R. Simoniello, M. Simonyan, P. Sinervo, N. B. Sinev, V. Sipica, G. Siragusa, A. Sircar, A. N. Sisakyan, S. Yu. Sivoklokov, J. Sjölin, T. B. Sjursen, H. P. Skottowe, K. Yu. Skovpen, P. Skubic, M. Slater, T. Slavicek, K. Sliwa, V. Smakhtin, B. H. Smart, L. Smestad, S. Yu. Smirnov, Y. Smirnov, L. N. Smirnova, O. Smirnova, M. Smizanska, K. Smolek, A. A. Snesarev, G. Snidero, J. Snow, S. Snyder, R. Sobie, F. Socher, J. Sodomka, A. Soffer, D. A. Soh, C. A. Solans, M. Solar, J. Solc, E. Yu. Soldatov, U. Soldevila, E. Solfaroli Camillocci, A. A. Solodkov, O. V. Solovyanov, V. Solovyev, P. Sommer, H. Y. Song, N. Soni, A. Sood, A. Sopczak, V. Sopko, B. Sopko, V. Sorin, M. Sosebee, R. Soualah, P. Soueid, A. M. Soukharev, D. South, S. Spagnolo, F. Spanò, W. R. Spearman, R. Spighi, G. Spigo, M. Spousta, T. Spreitzer, B. Spurlock, R. D. St. Denis, S. Staerz, J. Stahlman, R. Stamen, E. Stanecka, R. W. Stanek, C. Stanescu, M. Stanescu-Bellu, M. M. Stanitzki, S. Stapnes, E. A. Starchenko, J. Stark, P. Staroba, P. Starovoitov, R. Staszewski, P. Stavina, G. Steele, P. Steinberg, I. Stekl, B. Stelzer, H. J. Stelzer, O. Stelzer-Chilton, H. Stenzel, S. Stern, G. A. Stewart, J. A. Stillings, M. C. Stockton, M. Stoebe, G. Stoicea, P. Stolte, S. Stonjek, A. R. Stradling, A. Straessner, M. E. Stramaglia, J. Strandberg, S. Strandberg, A. Strandlie, E. Strauss, M. Strauss, P. Strizenec, R. Ströhmer, D. M. Strom, R. Stroynowski, S. A. Stucci, B. Stugu, N. A. Styles, D. Su, J. Su, HS. Subramania, R. Subramaniam, A. Succurro, Y. Sugaya, C. Suhr, M. Suk, V. V. Sulin, S. Sultansoy, T. Sumida, X. Sun, J. E. Sundermann, K. Suruliz, G. Susinno, M. R. Sutton, Y. Suzuki, M. Svatos, S. Swedish, M. Swiatlowski, I. Sykora, T. Sykora, D. Ta, K. Tackmann, J. Taenzer, A. Taffard, R. Tafirout, N. Taiblum, Y. Takahashi, H. Takai, R. Takashima, H. Takeda, T. Takeshita, Y. Takubo, M. Talby, A. A. Talyshev, J. Y. C. Tam, M. C. Tamsett, K. G. Tan, J. Tanaka, R. Tanaka, S. Tanaka, S. Tanaka, A. J. Tanasijczuk, K. Tani, N. Tannoury, S. Tapprogge, S. Tarem, F. Tarrade, G. F. Tartarelli, P. Tas, M. Tasevsky, T. Tashiro, E. Tassi, A. Tavares Delgado, Y. Tayalati, F. E. Taylor, G. N. Taylor, W. Taylor, F. A. Teischinger, M. Teixeira Dias Castanheira, P. Teixeira-Dias, K. K. Temming, H. Ten Kate, P. K. Teng, S. Terada, K. Terashi, J. Terron, S. Terzo, M. Testa, R. J. Teuscher, J. Therhaag, T. Theveneaux-Pelzer, S. Thoma, J. P. Thomas, J. Thomas-Wilsker, E. N. Thompson, P. D. Thompson, P. D. Thompson, A. S. Thompson, L. A. Thomsen, E. Thomson, M. Thomson, W. M. Thong, R. P. Thun, F. Tian, M. J. Tibbetts, V. O. Tikhomirov, Yu. A. Tikhonov, S. Timoshenko, E. Tiouchichine, P. Tipton, S. Tisserant, T. Todorov, S. Todorova-Nova, B. Toggerson, J. Tojo, S. Tokár, K. Tokushuku, K. Tollefson, L. Tomlinson, M. Tomoto, L. Tompkins, K. Toms, N. D. Topilin, E. Torrence, H. Torres, E. Torró Pastor, J. Toth, F. Touchard, D. R. Tovey, H. L. Tran, T. Trefzger, L. Tremblet, A. Tricoli, I. M. Trigger, S. Trincaz-Duvoid, M. F. Tripiana, N. Triplett, W. Trischuk, B. Trocmé, C. Troncon, M. Trottier-McDonald, M. Trovatelli, P. True, M. Trzebinski, A. Trzupek, C. Tsarouchas, J. C-L. Tseng, P. V. Tsiareshka, D. Tsionou, G. Tsipolitis, N. Tsirintanis, S. Tsiskaridze, V. Tsiskaridze, E. G. Tskhadadze, I. I. Tsukerman, V. Tsulaia, S. Tsuno, D. Tsybychev, A. Tudorache, V. Tudorache, A. N. Tuna, S. A. Tupputi, S. Turchikhin, D. Turecek, I. Turk Cakir, R. Turra, P. M. Tuts, A. Tykhonov, M. Tylmad, M. Tyndel, K. Uchida, I. Ueda, R. Ueno, M. Ughetto, M. Ugland, M. Uhlenbrock, F. Ukegawa, G. Unal, A. Undrus, G. Unel, F. C. Ungaro, Y. Unno, D. Urbaniec, P. Urquijo, G. Usai, A. Usanova, L. Vacavant, V. Vacek, B. Vachon, N. Valencic, S. Valentinetti, A. Valero, L. Valery, S. Valkar, E. Valladolid Gallego, S. Vallecorsa, J. A. Valls Ferrer, R. Van Berg, P. C. Van Der Deijl, R. van der Geer, H. van der Graaf, R. Van Der Leeuw, D. van der Ster, N. van Eldik, P. van Gemmeren, J. Van Nieuwkoop, I. van Vulpen, M. C. van Woerden, M. Vanadia, W. Vandelli, R. Vanguri, A. Vaniachine, P. Vankov, F. Vannucci, G. Vardanyan, R. Vari, E. W. Varnes, T. Varol, D. Varouchas, A. Vartapetian, K. E. Varvell, F. Vazeille, T. Vazquez Schroeder, J. Veatch, F. Veloso, S. Veneziano, A. Ventura, D. Ventura, M. Venturi, N. Venturi, A. Venturini, V. Vercesi, M. Verducci, W. Verkerke, J. C. Vermeulen, A. Vest, M. C. Vetterli, O. Viazlo, I. Vichou, T. Vickey, O. E. Vickey Boeriu, G. H. A. Viehhauser, S. Viel, R. Vigne, M. Villa, M. Villaplana Perez, E. Vilucchi, M. G. Vincter, V. B. Vinogradov, J. Virzi, I. Vivarelli, F. Vives Vaque, S. Vlachos, D. Vladoiu, M. Vlasak, A. Vogel, P. Vokac, G. Volpi, M. Volpi, H. von der Schmitt, H. von Radziewski, E. von Toerne, V. Vorobel, K. Vorobev, M. Vos, R. Voss, J. H. Vossebeld, N. Vranjes, M. Vranjes Milosavljevic, V. Vrba, M. Vreeswijk, T. Vu Anh, R. Vuillermet, I. Vukotic, Z. Vykydal, W. Wagner, P. Wagner, S. Wahrmund, J. Wakabayashi, J. Walder, R. Walker, W. Walkowiak, R. Wall, P. Waller, B. Walsh, C. Wang, C. Wang, F. Wang, H. Wang, H. Wang, J. Wang, J. Wang, K. Wang, R. Wang, S. M. Wang, T. Wang, X. Wang, C. Wanotayaroj, A. Warburton, C. P. Ward, D. R. Wardrope, M. Warsinsky, A. Washbrook, C. Wasicki, I. Watanabe, P. M. Watkins, A. T. Watson, I. J. Watson, M. F. Watson, G. Watts, S. Watts, B. M. Waugh, S. Webb, M. S. Weber, S. W. Weber, J. S. Webster, A. R. Weidberg, P. Weigell, B. Weinert, J. Weingarten, C. Weiser, H. Weits, P. S. Wells, T. Wenaus, D. Wendland, Z. Weng, T. Wengler, S. Wenig, N. Wermes, M. Werner, P. Werner, M. Wessels, J. Wetter, K. Whalen, A. White, M. J. White, R. White, S. White, D. Whiteson, D. Wicke, F. J. Wickens, W. Wiedenmann, M. Wielers, P. Wienemann, C. Wiglesworth, L. A. M. Wiik-Fuchs, P. A. Wijeratne, A. Wildauer, M. A. Wildt, H. G. Wilkens, J. Z. Will, H. H. Williams, S. Williams, C. Willis, S. Willocq, J. A. Wilson, A. Wilson, I. Wingerter-Seez, F. Winklmeier, M. Wittgen, T. Wittig, J. Wittkowski, S. J. Wollstadt, M. W. Wolter, H. Wolters, B. K. Wosiek, J. Wotschack, M. J. Woudstra, K. W. Wozniak, M. Wright, M. Wu, S. L. Wu, X. Wu, Y. Wu, E. Wulf, T. R. Wyatt, B. M. Wynne, S. Xella, M. Xiao, D. Xu, L. Xu, B. Yabsley, S. Yacoob, M. Yamada, H. Yamaguchi, Y. Yamaguchi, A. Yamamoto, K. Yamamoto, S. Yamamoto, T. Yamamura, T. Yamanaka, K. Yamauchi, Y. Yamazaki, Z. Yan, H. Yang, H. Yang, U. K. Yang, Y. Yang, S. Yanush, L. Yao, W-M. Yao, Y. Yasu, E. Yatsenko, K. H. Yau Wong, J. Ye, S. Ye, A. L. Yen, E. Yildirim, M. Yilmaz, R. Yoosoofmiya, K. Yorita, R. Yoshida, K. Yoshihara, C. Young, C. J. S. Young, S. Youssef, D. R. Yu, J. Yu, J. M. Yu, J. Yu, L. Yuan, A. Yurkewicz, B. Zabinski, R. Zaidan, A. M. Zaitsev, A. Zaman, S. Zambito, L. Zanello, D. Zanzi, A. Zaytsev, C. Zeitnitz, M. Zeman, A. Zemla, K. Zengel, O. Zenin, T. Ženiš, D. Zerwas, G. Zevi della Porta, D. Zhang, F. Zhang, H. Zhang, J. Zhang, L. Zhang, X. Zhang, Z. Zhang, Z. Zhao, A. Zhemchugov, J. Zhong, B. Zhou, L. Zhou, N. Zhou, C. G. Zhu, H. Zhu, J. Zhu, Y. Zhu, X. Zhuang, A. Zibell, D. Zieminska, N. I. Zimine, C. Zimmermann, R. Zimmermann, S. Zimmermann, S. Zimmermann, Z. Zinonos, M. Ziolkowski, G. Zobernig, A. Zoccoli, M. zur Nedden, G. Zurzolo, V. Zutshi, L. Zwalinski, The ATLAS Collaboration

**Affiliations:** 1 Department of Physics, University of Adelaide, Adelaide, Australia; 2 Physics Department, SUNY Albany, Albany, NY USA; 3 Department of Physics, University of Alberta, Edmonton, AB Canada; 4 Department of Physics, Ankara University, Ankara, Turkey; Department of Physics, Gazi University, Ankara, Turkey; Division of Physics, TOBB University of Economics and Technology, Ankara, Turkey; Turkish Atomic Energy Authority, Ankara, Turkey; 5 LAPP, CNRS/IN2P3 and Université de Savoie, Annecy-le-Vieux, France; 6 High Energy Physics Division, Argonne National Laboratory, Argonne, IL USA; 7 Department of Physics, University of Arizona, Tucson, AZ USA; 8 Department of Physics, The University of Texas at Arlington, Arlington, TX USA; 9 Physics Department, University of Athens, Athens, Greece; 10 Physics Department, National Technical University of Athens, Zografou, Greece; 11 Institute of Physics, Azerbaijan Academy of Sciences, Baku, Azerbaijan; 12 Institut de Física d’Altes Energies and Departament de Física de la Universitat Autònoma de Barcelona, Barcelona, Spain; 13 Institute of Physics, University of Belgrade, Belgrade, Serbia; Vinca Institute of Nuclear Sciences, University of Belgrade, Belgrade, Serbia; 14 Department for Physics and Technology, University of Bergen, Bergen, Norway; 15 Physics Division, Lawrence Berkeley National Laboratory and University of California, Berkeley, CA USA; 16 Department of Physics, Humboldt University, Berlin, Germany; 17 Albert Einstein Center for Fundamental Physics and Laboratory for High Energy Physics, University of Bern, Bern, Switzerland; 18 School of Physics and Astronomy, University of Birmingham, Birmingham, UK; 19 Department of Physics, Bogazici University, Istanbul, Turkey; Department of Physics, Dogus University, Istanbul, Turkey; Department of Physics Engineering, Gaziantep University, Gaziantep, Turkey; 20 INFN Sezione di Bologna, Bologna, Italy; Dipartimento di Fisica e Astronomia, Università di Bologna, Bologna, Italy; 21 Physikalisches Institut, University of Bonn, Bonn, Germany; 22 Department of Physics, Boston University, Boston, MA USA; 23 Department of Physics, Brandeis University, Waltham, MA USA; 24 Universidade Federal do Rio De Janeiro COPPE/EE/IF, Rio de Janeiro, Brazil; Federal University of Juiz de Fora (UFJF), Juiz de Fora, Brazil; Federal University of Sao Joao del Rei (UFSJ), Sao Joao del Rei, Brazil; Instituto de Fisica, Universidade de Sao Paulo, Sao Paulo, Brazil; 25 Physics Department, Brookhaven National Laboratory, Upton, NY USA; 26 National Institute of Physics and Nuclear Engineering, Bucharest, Romania; Physics Department, National Institute for Research and Development of Isotopic and Molecular Technologies, Cluj Napoca, Romania; University Politehnica Bucharest, Bucharest, Romania; West University in Timisoara, Timisoara, Romania; 27 Departamento de Física, Universidad de Buenos Aires, Buenos Aires, Argentina; 28 Cavendish Laboratory, University of Cambridge, Cambridge, UK; 29 Department of Physics, Carleton University, Ottawa, ON Canada; 30 CERN, Geneva, Switzerland; 31 Enrico Fermi Institute, University of Chicago, Chicago, IL USA; 32 Departamento de Física, Pontificia Universidad Católica de Chile, Santiago, Chile; Departamento de Física, Universidad Técnica Federico Santa María, Valparaiso, Chile; 33 Institute of High Energy Physics, Chinese Academy of Sciences, Beijing, China; Department of Modern Physics, University of Science and Technology of China, Hefei, Anhui, China; Department of Physics, Nanjing University, Nanjing, Jiangsu, China; School of Physics, Shandong University, Jinan, Shandong, China; Physics Department, Shanghai Jiao Tong University, Shanghai, China; 34 Laboratoire de Physique Corpusculaire, Clermont Université and Université Blaise Pascal and CNRS/IN2P3, Clermont-Ferrand, France; 35 Nevis Laboratory, Columbia University, Irvington, NY USA; 36 Niels Bohr Institute, University of Copenhagen, Copenhagen, Denmark; 37 INFN Gruppo Collegato di Cosenza, Laboratori Nazionali di Frascati, Frascati, Italy; Dipartimento di Fisica, Università della Calabria, Rende, Italy; 38 Faculty of Physics and Applied Computer Science, AGH University of Science and Technology, Kraków, Poland; Marian Smoluchowski Institute of Physics, Jagiellonian University, Kraków, Poland; 39 The Henryk Niewodniczanski Institute of Nuclear Physics, Polish Academy of Sciences, Kraków, Poland; 40 Physics Department, Southern Methodist University, Dallas, TX USA; 41 Physics Department, University of Texas at Dallas, Richardson, TX USA; 42 DESY, Hamburg and Zeuthen, Germany; 43 Institut für Experimentelle Physik IV, Technische Universität Dortmund, Dortmund, Germany; 44 Institut für Kern- und Teilchenphysik, Technische Universität Dresden, Dresden, Germany; 45 Department of Physics, Duke University, Durham, NC USA; 46 SUPA-School of Physics and Astronomy, University of Edinburgh, Edinburgh, UK; 47 INFN Laboratori Nazionali di Frascati, Frascati, Italy; 48 Fakultät für Mathematik und Physik, Albert-Ludwigs-Universität, Freiburg, Germany; 49 Section de Physique, Université de Genève, Geneva, Switzerland; 50 INFN Sezione di Genova, Genoa, Italy; Dipartimento di Fisica, Università di Genova, Genova, Italy; 51 E. Andronikashvili Institute of Physics, Iv. Javakhishvili Tbilisi State University, Tbilisi, Georgia; High Energy Physics Institute, Tbilisi State University, Tbilisi, Georgia; 52 II Physikalisches Institut, Justus-Liebig-Universität Giessen, Giessen, Germany; 53 SUPA-School of Physics and Astronomy, University of Glasgow, Glasgow, UK; 54 II Physikalisches Institut, Georg-August-Universität, Göttingen, Germany; 55 Laboratoire de Physique Subatomique et de Cosmologie, Université Grenoble-Alpes, CNRS/IN2P3, Grenoble, France; 56 Department of Physics, Hampton University, Hampton, VA USA; 57 Laboratory for Particle Physics and Cosmology, Harvard University, Cambridge, MA USA; 58 Kirchhoff-Institut für Physik, Ruprecht-Karls-Universität Heidelberg, Heidelberg, Germany; Physikalisches Institut, Ruprecht-Karls-Universität Heidelberg, Heidelberg, Germany; ZITI Institut für technische Informatik, Ruprecht-Karls-Universität Heidelberg, Mannheim, Germany; 59 Faculty of Applied Information Science, Hiroshima Institute of Technology, Hiroshima, Japan; 60 Department of Physics, Indiana University, Bloomington, IN USA; 61 Institut für Astro- und Teilchenphysik, Leopold-Franzens-Universität, Innsbruck, Austria; 62 University of Iowa, Iowa City, IA USA; 63 Department of Physics and Astronomy, Iowa State University, Ames, IA USA; 64 Joint Institute for Nuclear Research, JINR Dubna, Dubna, Russia; 65 KEK, High Energy Accelerator Research Organization, Tsukuba, Japan; 66 Graduate School of Science, Kobe University, Kobe, Japan; 67 Faculty of Science, Kyoto University, Kyoto, Japan; 68 Kyoto University of Education, Kyoto, Japan; 69 Department of Physics, Kyushu University, Fukuoka, Japan; 70 Instituto de Física La Plata, Universidad Nacional de La Plata and CONICET, La Plata, Argentina; 71 Physics Department, Lancaster University, Lancaster, UK; 72 INFN Sezione di Lecce, Lecce, Italy; Dipartimento di Matematica e Fisica, Università del Salento, Lecce, Italy; 73 Oliver Lodge Laboratory, University of Liverpool, Liverpool, UK; 74 Department of Physics, Jožef Stefan Institute and University of Ljubljana, Ljubljana, Slovenia; 75 School of Physics and Astronomy, Queen Mary University of London, London, UK; 76 Department of Physics, Royal Holloway University of London, Surrey, UK; 77 Department of Physics and Astronomy, University College London, London, UK; 78 Louisiana Tech University, Ruston, LA USA; 79 Laboratoire de Physique Nucléaire et de Hautes Energies, UPMC and Université Paris-Diderot and CNRS/IN2P3, Paris, France; 80 Fysiska institutionen, Lunds universitet, Lund, Sweden; 81 Departamento de Fisica Teorica C-15, Universidad Autonoma de Madrid, Madrid, Spain; 82 Institut für Physik, Universität Mainz, Mainz, Germany; 83 School of Physics and Astronomy, University of Manchester, Manchester, UK; 84 CPPM, Aix-Marseille Université and CNRS/IN2P3, Marseille, France; 85 Department of Physics, University of Massachusetts, Amherst, MA USA; 86 Department of Physics, McGill University, Montreal, QC Canada; 87 School of Physics, University of Melbourne, Parkville, VIC Australia; 88 Department of Physics, The University of Michigan, Ann Arbor, MI USA; 89 Department of Physics and Astronomy, Michigan State University, East Lansing, MI USA; 90 INFN Sezione di Milano, Milan, Italy; Dipartimento di Fisica, Università di Milano, Milan, Italy; 91 B.I. Stepanov Institute of Physics, National Academy of Sciences of Belarus, Minsk, Republic of Belarus; 92 National Scientific and Educational Centre for Particle and High Energy Physics, Minsk, Republic of Belarus; 93 Department of Physics, Massachusetts Institute of Technology, Cambridge, MA USA; 94 Group of Particle Physics, University of Montreal, Montreal, QC Canada; 95 P.N. Lebedev Institute of Physics, Academy of Sciences, Moscow, Russia; 96 Institute for Theoretical and Experimental Physics (ITEP), Moscow, Russia; 97 Moscow Engineering and Physics Institute (MEPhI), Moscow, Russia; 98 D.V. Skobeltsyn Institute of Nuclear Physics, M.V. Lomonosov Moscow State University, Moscow, Russia; 99 Fakultät für Physik, Ludwig-Maximilians-Universität München, Munich, Germany; 100 Max-Planck-Institut für Physik (Werner-Heisenberg-Institut), Munich, Germany; 101 Nagasaki Institute of Applied Science, Nagasaki, Japan; 102 Graduate School of Science and Kobayashi-Maskawa Institute, Nagoya University, Nagoya, Japan; 103 INFN Sezione di Napoli, Naples, Italy; Dipartimento di Fisica, Università di Napoli, Naples, Italy; 104 Department of Physics and Astronomy, University of New Mexico, Albuquerque, NM USA; 105 Institute for Mathematics, Astrophysics and Particle Physics, Radboud University Nijmegen/Nikhef, Nijmegen, Netherlands; 106 Nikhef National Institute for Subatomic Physics and University of Amsterdam, Amsterdam, The Netherlands; 107 Department of Physics, Northern Illinois University, DeKalb, IL USA; 108 Budker Institute of Nuclear Physics, SB RAS, Novosibirsk, Russia; 109 Department of Physics, New York University, New York, NY USA; 110 Ohio State University, Columbus, OH USA; 111 Faculty of Science, Okayama University, Okayama, Japan; 112 Homer L. Dodge Department of Physics and Astronomy, University of Oklahoma, Norman, OK USA; 113 Department of Physics, Oklahoma State University, Stillwater, OK USA; 114 Palacký University, RCPTM, Olomouc, Czech Republic; 115 Center for High Energy Physics, University of Oregon, Eugene, OR USA; 116 LAL, Université Paris-Sud and CNRS/IN2P3, Orsay, France; 117 Graduate School of Science, Osaka University, Osaka, Japan; 118 Department of Physics, University of Oslo, Oslo, Norway; 119 Department of Physics, Oxford University, Oxford, UK; 120 INFN Sezione di Pavia, Pavia, Italy; Dipartimento di Fisica, Università di Pavia, Pavia, Italy; 121 Department of Physics, University of Pennsylvania, Philadelphia, PA USA; 122 Petersburg Nuclear Physics Institute, Gatchina, Russia; 123 INFN Sezione di Pisa, Pisa, Italy; Dipartimento di Fisica E. Fermi, Università di Pisa, Pisa, Italy; 124 Department of Physics and Astronomy, University of Pittsburgh, Pittsburgh, PA USA; 125 Laboratorio de Instrumentacao e Fisica Experimental de Particulas-LIP, Lisbon, Portugal; Faculdade de Ciências, Universidade de Lisboa, Lisbon, Portugal; Department of Physics, University of Coimbra, Coimbra, Portugal; Centro de Física Nuclear da Universidade de Lisboa, Lisbon, Portugal; Departamento de Fisica, Universidade do Minho, Braga, Portugal; Departamento de Fisica Teorica y del Cosmos and CAFPE, Universidad de Granada, Granada, Spain; Dep Fisica and CEFITEC of Faculdade de Ciencias e Tecnologia, Universidade Nova de Lisboa, Caparica, Portugal; 126 Institute of Physics, Academy of Sciences of the Czech Republic, Prague, Czech Republic; 127 Czech Technical University in Prague, Prague, Czech Republic; 128 Faculty of Mathematics and Physics, Charles University in Prague, Prague, Czech Republic; 129 State Research Center Institute for High Energy Physics, Protvino, Russia; 130 Particle Physics Department, Rutherford Appleton Laboratory, Didcot, UK; 131 Physics Department, University of Regina, Regina, SK Canada; 132 Ritsumeikan University, Kusatsu, Shiga Japan; 133 INFN Sezione di Roma, Rome, Italy; Dipartimento di Fisica, Sapienza Università di Roma, Rome, Italy; 134 INFN Sezione di Roma Tor Vergata, Rome, Italy; Dipartimento di Fisica, Università di Roma Tor Vergata, Rome, Italy; 135 INFN Sezione di Roma Tre, Rome, Italy; Dipartimento di Matematica e Fisica, Università Roma Tre, Rome, Italy; 136 Faculté des Sciences Ain Chock, Réseau Universitaire de Physique des Hautes Energies-Université Hassan II, Casablanca, Morocco; Centre National de l’Energie des Sciences Techniques Nucleaires, Rabat, Morocco; Faculté des Sciences Semlalia, Université Cadi Ayyad, LPHEA-Marrakech, Marrakech, Morocco; Faculté des Sciences, Université Mohamed Premier and LPTPM, Oujda, Morocco; Faculté des Sciences, Université Mohammed V-Agdal, Rabat, Morocco; 137 DSM/IRFU (Institut de Recherches sur les Lois Fondamentales de l’Univers), CEA Saclay (Commissariat à l’Energie Atomique et aux Energies Alternatives), Gif-sur-Yvette, France; 138 Santa Cruz Institute for Particle Physics, University of California Santa Cruz, Santa Cruz, CA USA; 139 Department of Physics, University of Washington, Seattle, WA USA; 140 Department of Physics and Astronomy, University of Sheffield, Sheffield, UK; 141 Department of Physics, Shinshu University, Nagano, Japan; 142 Fachbereich Physik, Universität Siegen, Siegen, Germany; 143 Department of Physics, Simon Fraser University, Burnaby, BC Canada; 144 SLAC National Accelerator Laboratory, Stanford, CA USA; 145 Faculty of Mathematics, Physics and Informatics, Comenius University, Bratislava, Slovak Republic; Department of Subnuclear Physics, Institute of Experimental Physics of the Slovak Academy of Sciences, Kosice, Slovak Republic; 146 Department of Physics, University of Cape Town, Cape Town, South Africa; Department of Physics, University of Johannesburg, Johannesburg, South Africa; School of Physics, University of the Witwatersrand, Johannesburg, South Africa; 147 Department of Physics, Stockholm University, Stockholm, Sweden; The Oskar Klein Centre, Stockholm, Sweden; 148 Physics Department, Royal Institute of Technology, Stockholm, Sweden; 149 Departments of Physics and Astronomy and Chemistry, Stony Brook University, Stony Brook, NY USA; 150 Department of Physics and Astronomy, University of Sussex, Brighton, UK; 151 School of Physics, University of Sydney, Sydney, Australia; 152 Institute of Physics, Academia Sinica, Taipei, Taiwan; 153 Department of Physics, Technion: Israel Institute of Technology, Haifa, Israel; 154 Raymond and Beverly Sackler School of Physics and Astronomy, Tel Aviv University, Tel Aviv, Israel; 155 Department of Physics, Aristotle University of Thessaloniki, Thessaloniki, Greece; 156 International Center for Elementary Particle Physics and Department of Physics, The University of Tokyo, Tokyo, Japan; 157 Graduate School of Science and Technology, Tokyo Metropolitan University, Tokyo, Japan; 158 Department of Physics, Tokyo Institute of Technology, Tokyo, Japan; 159 Department of Physics, University of Toronto, Toronto, ON Canada; 160 TRIUMF, Vancouver, BC, Canada; Department of Physics and Astronomy, York University, Toronto, ON Canada; 161 Faculty of Pure and Applied Sciences, University of Tsukuba, Tsukuba, Japan; 162 Department of Physics and Astronomy, Tufts University, Medford, MA USA; 163 Centro de Investigaciones, Universidad Antonio Narino, Bogota, Colombia; 164 Department of Physics and Astronomy, University of California Irvine, Irvine, CA USA; 165 INFN Gruppo Collegato di Udine, Sezione di Trieste, Udine, Italy; ICTP, Trieste, Italy; Dipartimento di Chimica, Fisica e Ambiente, Università di Udine, Udine, Italy; 166 Department of Physics, University of Illinois, Urbana, IL USA; 167 Department of Physics and Astronomy, University of Uppsala, Uppsala, Sweden; 168 Instituto de Física Corpuscular (IFIC) and Departamento de Física Atómica, Molecular y Nuclear and Departamento de Ingeniería Electrónica and Instituto de Microelectrónica de Barcelona (IMB-CNM), University of Valencia and CSIC, Valencia, Spain; 169 Department of Physics, University of British Columbia, Vancouver, BC Canada; 170 Department of Physics and Astronomy, University of Victoria, Victoria, BC Canada; 171 Department of Physics, University of Warwick, Coventry, UK; 172 Waseda University, Tokyo, Japan; 173 Department of Particle Physics, The Weizmann Institute of Science, Rehovot, Israel; 174 Department of Physics, University of Wisconsin, Madison, WI USA; 175 Fakultät für Physik und Astronomie, Julius-Maximilians-Universität, Würzburg, Germany; 176 Fachbereich C Physik, Bergische Universität Wuppertal, Wuppertal, Germany; 177 Department of Physics, Yale University, New Haven, CT USA; 178 Yerevan Physics Institute, Yerevan, Armenia; 179 Centre de Calcul de l’Institut National de Physique Nucléaire et de Physique des Particules (IN2P3), Villeurbanne, France; 180 CERN, 1211 Geneva 23, Switzerland

## Abstract

A likelihood-based discriminant for the identification of quark- and gluon-initiated jets is built and validated using 4.7 fb$^{-1}$ of proton–proton collision data at $\sqrt{s}=7$ $\mathrm {\ TeV}$ collected with the ATLAS detector at the LHC. Data samples with enriched quark or gluon content are used in the construction and validation of templates of jet properties that are the input to the likelihood-based discriminant. The discriminating power of the jet tagger is established in both data and Monte Carlo samples within a systematic uncertainty of $\approx$ 10–20 %. In data, light-quark jets can be tagged with an efficiency of $\approx 50\,\%$ while achieving a gluon-jet mis-tag rate of $\approx 25\,\%$ in a $p_{\mathrm {T}}$ range between $40\mathrm {\ GeV}$ and $360\mathrm {\ GeV}$ for jets in the acceptance of the tracker. The rejection of gluon-jets found in the data is significantly below what is attainable using a Pythia 6 Monte Carlo simulation, where gluon-jet mis-tag rates of 10 % can be reached for a 50 % selection efficiency of light-quark jets using the same jet properties.

## Introduction

The production of quarks and gluons via strong interactions is the dominant high-momentum-transfer process at the LHC and is a significant background to most new-physics searches. These partons are measured as jets, which are collimated streams of charged and neutral particles, clustered using dedicated algorithms. Corrections to measured quantities are necessary to relate the jets to their parent partons. Many gluons are generated in most common Standard Model processes, such as the inclusive production of jets [1, 2]. On the other hand, some processes arising from new-physics models, for example supersymmetry, generate many light quarks [3, 4]. The power to discriminate between jets initiated by light quarks and those initiated by gluons would therefore provide a powerful tool in searches for new physics. In case of a discovery of a new particle, such a discriminant could provide valuable information about its nature. Also, some Standard Model measurements rely on the correct identification of the origin of jets, as in the cases of reconstructing a hadronic $W$ decay when measuring the top quark mass, or in the reconstruction of a hadronic $Z$ decay when measuring the Higgs boson mass via $h\rightarrow ZZ\rightarrow \ell \ell q\bar{q}$. These analyses would benefit from such a discriminant. These applications motivate the analysis of the partonic origin of jets that is the focus of this paper.

In perturbative quantum chromodynamics (QCD), the concept of a parton initiating a jet is a fixed-order notion. In the matrix-element calculation of a high-momentum-transfer-process, the outgoing partons appear naïvely much like outgoing particles in the final state. However, only colourless states with two or more partons can form an observable jet. Moreover, in a parton shower, the leading parton is only well defined for a fixed number of splittings. The next step in the shower may change the energy, direction, or flavour of the leading parton. Thus, labelling jets with a specific flavour and interpreting results after such labelling requires a clearly defined procedure [5].

Certain parton branchings can yield an ambiguous jet identity. The labelling of a jet may also depend on the physics goal of the analysis. For example, a jet from the $q\bar{q}'$ decay of a high-momentum $W$ boson produced in a top quark decay can be considered either as a part of a top-quark jet or as a boosted $W$-boson jet. Nonetheless, many event topologies lend themselves to the identification of a jet as having originated from a specific type of parton in the matrix-element calculation. Such an approach can lead to an unambiguous and meaningful parton labelling for a large majority of jets. This approach of linking jet-by-jet labelling to the results of the underlying leading-order (LO) calculation is also used in this paper to define the flavour of a jet.

Discrimination between jets of different partonic origin has been attempted previously at several experiments [6–16]. Most work has relied on jet properties that result from the difference in colour charge between the partons. The colour factors in quantum chromodynamics differ for quarks ($C_F=4/3$) and gluons ($C_A=3$), and therefore, for example, one expects approximately $C_A/C_F=9/4$ times more particles in a gluon-initiated jet than in a jet initiated by a light ($u$, $d$ or $s$) quark. The measured difference in particle multiplicity at OPAL was, in fact, not far from this expectation [9]. Because of the showering that produces these additional particles, gluon jets are also expected to be wider and have a softer particle spectrum.

The most successful studies of discrimination between light-quark-initiated and gluon-initiated jets (henceforth, quark-jets and gluon-jets) have taken place at electron-positron colliders [17, 18]. The selection and identification of “pure” samples of quark- and gluon-jets is considerably more difficult at hadron colliders because of the complication added by beam remnants, initial-state radiation, and multi-parton interactions. The presence of multiple soft $pp$ collisions overlaying the hard-scatter interaction of interest at the LHC further complicates this task. Recently some effort has been devoted to developing kinematic selections that significantly enhance the fraction of quark-jets or gluon-jets in a set of events [5]. In addition, discriminants based on jet structure have shown some promise for distinguishing between classes of jets at the LHC [19].

Jets that include, or are initiated by, heavy quarks (bottom and charm) also exhibit properties different from those of quark-jets [20, 21]. Generally, these jets are wider than quark-jets. They are often identified by long-lived or leptonically decaying hadrons. However, no special discriminant for them is developed here.

This paper is organised as follows. The ATLAS detector is briefly described in Sect. 2. Section 3 describes details of the data and Monte Carlo (MC) samples used, as well as the object reconstruction and event selection. Section 4 introduces the definition of gluon-jets and quark-jets that are used in the remainder of the paper. The jet properties used to build a discriminant from samples with different purities, and the validation of the extraction method using MC event samples, are described in Sect. 5. Section 6 describes the selection of samples based on kinematic variables to enhance quark-jet or gluon-jet fractions and the validation of the extracted properties using those samples. The likelihood-based discriminant is described in Sect. 7, where its performance in MC simulation and in data is discussed. Finally, the conclusions are presented in Sect. 8.

## ATLAS detector

The ATLAS detector [22] comprises an inner tracking detector, a calorimeter system, and a muon spectrometer. The inner detector (ID) includes a silicon pixel detector, a silicon microstrip detector and a transition radiation tracker. It is immersed in a 2 T axial magnetic field provided by a solenoid and precisely measures the trajectories of charged particles with $|\eta |<2.5$.^1^ The calorimeter system covers the region $|\eta |<4.9$ and is divided into electromagnetic and hadronic compartments. Electromagnetic calorimetry in the region $|\eta |<3.2$ is provided by liquid-argon sampling calorimeters with lead absorbers. In the barrel region ($|\eta |<1.7$), the hadronic calorimeter comprises scintillator tiles with steel absorbers, and the endcap region ($1.4<|\eta |<3.2$) is covered by a liquid-argon and copper sampling hadronic calorimeter. The calorimetry in the forward region ($3.2<|\eta |<4.9$) is provided by a liquid-argon and copper sampling electromagnetic calorimeter and a liquid-argon and tungsten sampling hadronic calorimeter. The muon spectrometer (MS) covers $|\eta |<2.7$ and uses a system of air-core toroidal magnets.

ATLAS has a three-level trigger system to select events. The first-level trigger uses custom-built hardware components and identifies jet, electron and photon candidates using coarse calorimeter information, and muon candidates using coarse tracking information from the muon spectrometer. At the highest level, full event reconstruction, similar to that used in the offline software, is performed to accurately identify and measure objects that determine whether the event is recorded.

## Data sample and event selection

Several samples are used in the construction and validation of the variables entering the quark/gluon discriminant: dijet events, trijet events, $\gamma$+jet events, $\gamma$+2-jet events, $t\bar{t}$ events and $W$+jet events. After basic data quality requirements are imposed to remove known detector errors and readout problems, the selected dataset corresponds to a total integrated luminosity of $4.67 \pm 0.08\;\text {fb}^{-1}$ [23]. The data were collected from March to October 2011 at a centre-of-mass energy $\sqrt{s}=7\mathrm {\ TeV}$. The average number of additional $pp$ collisions per bunch crossing, called “pile-up”, rose during the data-taking period from a few to 15.

### Monte Carlo simulation

Simulated event samples are generated for comparison with data and for the determination of the systematic uncertainties based on variations in the MC generator settings. For the MC samples, several different generators are used. MadGraph [24] is run as a $2\rightarrow N$ generator with MLM matching [25], uses the CTEQ 6L1 parton distribution function (PDF) set, and is interfaced to Pythia 6 with a version of the ATLAS MC11 Underlying Event Tune 2B (AUET2B) [26] constructed for this PDF set. Herwig++ [27] is run standalone as a $2\rightarrow 2$ generator and uses the MRST LO** PDF set with the LHC-UE7-2 tune [28]. This tune of Herwig++ has an improved description of colour reconnection in multiple parton interactions and has been shown to have fair agreement with ATLAS data in minimum-bias observables [28]. Pythia 6 is also run standalone as a $2\rightarrow 2$ generator with the MRST LO** PDF set and the AUET2B tune. The AUET2B tune incorporates ATLAS [29] and CDF [30] jet-shape measurements as well as ATLAS fragmentation function measurements at $\sqrt{s}=7$ $\mathrm {\ TeV}$ [31] and is thus expected to describe inclusive-jet properties well.

Additional pile-up events, which are superimposed on the hard-scattering event, are generated with either Pythia 6 [32] with the AUET2B tune using the MRST LO** PDF [33] set, or Pythia 8 [34] with the 4C tune [35] using the CTEQ 6L1 PDF set [36]. Choosing between these two pile-up simulations has negligible impact on the analysis. The number of pile-up events in the MC simulation is reweighted to match the conditions found in the data for each trigger selection. The events are passed through the ATLAS detector simulation [37], based on GEANT4 [38], and are reconstructed using the same software as for the data.

### Jet reconstruction, selection and calibration

Jets are constructed from topological clusters of calorimeter cells [39] and calibrated using the EM+JES scheme [1, 40]. This scheme is designed to adjust the energy measured in the calorimeter to that of the true particle jets on average. Calorimeter jets are reconstructed using the anti-$k_{t}$ jet algorithm [41, 42] with a four-momentum recombination scheme and studied if calibrated transverse momentum $p_{\mathrm {T}}>20$ $\mathrm {\ GeV}$ and $|\eta |<4.5$. Jet-finding radius parameters of both $R=0.4$ and $R=0.6$ are studied. Only jets with $|\eta |<2.1$ are used for building the quark-jet tagger, to guarantee that the jet is well within the tracking acceptance. In the MC simulation, particle jets are reconstructed using the same anti-$k_{t}$ algorithm with stable, interacting particles^2^ as input to the jet algorithm. In all cases, jet finding is done in $(\mathrm{rapidity},\phi )$ coordinates and jet calibration is done in $(\eta ^\mathrm{jet},\phi ^\mathrm{\ jet})$ coordinates.

The reconstructed jets are additionally required to satisfy several data quality and isolation criteria. The data quality cuts are each designed to mitigate the impact of specific non-collision backgrounds [1]. Reconstructed and particle jets are considered isolated if there is no other reconstructed jet (or particle jet) within a cone of size $\Delta R =\sqrt{(\Delta \eta )^2+(\Delta \phi )^2}<0.7$ around the jet axis. Only isolated jets are considered in this study. The jet vertex fraction (JVF) is calculated for each jet and used to reject jets originating from pile-up interactions. The JVF is built using information about the origin, along the direction of the beam, of tracks with $\Delta R <0.4$ ($\Delta R <0.6$) to the jet axis for $R=0.4$ ($R=0.6$) jets and describes the fraction of the jet’s charged particle $p_{\mathrm {T}}$ associated with the primary vertex [40].

### Track selection and associating tracks with jets

Tracks are associated with jets by requiring that the track momentum direction (calculated at the primary vertex) and the jet direction satisfy $\Delta R (\mathrm{jet,track})<0.4$ ($\Delta R (\mathrm{jet,track}) <0.6$) for $R=0.4$ ($R=0.6$) jets. Track parameters are evaluated at the point of closest approach to the primary hard-scattering vertex, which is the vertex with the highest sum of associated track $p_{\mathrm {T}}^2$. Tracks are required to have $p_{\mathrm {T}}>1$ $\mathrm {\ GeV}$, at least one pixel hit and at least six hits in the silicon strip tracker, as well as transverse (longitudinal) impact parameters with respect to the hard-scattering vertex $|d_0|<1$ mm ($|z_0\cdot \sin (\theta )|<1$ mm).

The studies in this paper were also performed with a requirement of track $p_{\mathrm {T}}>500$ $\mathrm {\ MeV}$. No significant changes to the results were found. Requiring $p_{\mathrm {T}}>1$ $\mathrm {\ GeV}$ reduces the sensitivity to pile-up and the underlying event, and this requirement is used for the remainder of the paper. A “ghost association” [43] procedure was also tested in place of $\Delta R$-based matching, and no significant differences are observed. The jet isolation requirement helps to guarantee the similarity of the ghost association procedure and the $\Delta R$ matching.

### Photon selection

Photons with $p_{\mathrm {T}}>25$ $\mathrm {\ GeV}$ are selected with pseudorapidity $|\eta |<2.37$, excluding the transition region between the barrel and end-cap calorimeters ($1.37<|\eta |<1.52$). Only the leading photon in the event is considered. The photons are required to satisfy the preselection and “tight” photon cuts described in Ref. [44]. An additional isolation cut requiring less than $5\mathrm {\ GeV}$ of transverse energy in a cone of size $\Delta R =0.4$ around the photon is imposed to increase the purity of the sample [40]. The photons are additionally required to be well separated from calorimeter defects and to not be within $\Delta R <0.4$ of a jet arising from non-collision backgrounds or out-of-time pile-up.

### Lepton selection

Isolated electrons and muons are used to select $W+$jet and $t\bar{t}$ events. Electron candidates are formed by matching clusters found in the electromagnetic calorimeter to tracks reconstructed in the ID in the region $|\eta |< 2.47$ and are required to have transverse energy $E_{\mathrm {T}}> 25 \mathrm {\ GeV}$. To ensure good containment of electromagnetic showers in the calorimeter, the transition region $1.37 <|\eta | < 1.52$ is excluded as for photons. The electron candidates must pass the “tight” selection criteria based on the lateral and transverse shapes of the clusters described in Ref. [45] but updated for 2011 running conditions. Reconstructed tracks in the ID and the MS are combined to form muon candidates, which are selected in the region $|\eta |<2.5$ and are required to have $p_{\mathrm {T}}> 20 \mathrm {\ GeV}$. The selection efficiency for electrons and muons in simulated events, as well as their energy and momentum scale and resolution, are adjusted to reproduce those observed in $Z\rightarrow \ell \ell$ events in data [45]. To reduce the contamination from jets identified as leptons, requirements are placed on the total momentum carried by tracks within $\Delta R =0.3$ of the lepton and on calorimeter energy deposits within $\Delta R =0.2$, excluding the track and energy of the lepton itself. For muons, the scalar sum of the $p_{\mathrm {T}}$ of these neighbouring tracks must be less than $2.5 \mathrm {\ GeV}$, while the sum of this close-by calorimeter $E_{\mathrm {T}}$ must be less than $4 \mathrm {\ GeV}$. For electrons, the sum of calorimeter $E_{\mathrm {T}}$ must be less than $6 \mathrm {\ GeV}$. Additionally, leptons are required to be consistent with originating from the primary hard-scattering vertex. They are required to have $|z_0|<10$ mm, and the ratio of $d_0$ to its uncertainty ($d_0$ significance) must be smaller than 3.0 for muons and 10.0 for electrons, due to the wider distribution found in signal electrons caused by bremsstrahlung.

### Trigger and event selection

All events must have a vertex with at least three associated tracks with $p_{\mathrm {T}}>150$ $\mathrm {\ MeV}$. Other event selection requirements are described below.

#### Dijet and trijet samples

The dijet sample is selected using single-jet triggers with various thresholds [46], which are fully efficient for jets with $p_{\mathrm {T}}>40$ $\mathrm {\ GeV}$. Each jet $p_{\mathrm {T}}$ bin is filled exclusively by a single trigger that is fully efficient for jets in that $p_{\mathrm {T}}$ range, following Ref. [1]. The trijet sample uses the same trigger selection as the dijet sample. This guarantees that studies using the jet with the third highest $p_{\mathrm {T}}$ in each event are not biased by the trigger.

#### $\gamma$+jet and $\gamma$+2-jet samples

The $\gamma \text {+jet}$ sample is selected using single-photon triggers. The lowest threshold single-photon trigger is fully efficient for photons with $p_{\mathrm {T}}>25$ $\mathrm {\ GeV}$. For this sample, a back-to-back requirement for the photon and the leading jet, $\Delta \phi >2.8$, is imposed. An additional veto on soft radiation is also applied to further reduce background contamination [40]: the uncalibrated $p_{\mathrm {T}}$ of the sub-leading jet is required to be less than 30 % of the photon $p_{\mathrm {T}}$. Relying on the $p_{\mathrm {T}}$ balance of the photon and jet, each jet $p_{\mathrm {T}}$ bin is filled exclusively by a single-photon trigger that provides a fully efficient selection.

The same triggers are used in the $\gamma$+2-jet sample in each region of jet $p_{\mathrm {T}}$. Since the sub-leading jet $p_{\mathrm {T}}$ is lower than that of the leading jet by definition, this selection is also not biased by jet reconstruction effects.

#### $W+$jet sample

The $W+$jet sample is selected using a single-electron or single-muon trigger. The event selection, following Ref. [47], requires exactly one charged lepton (electron or muon) and that it matches the trigger accepting the event, a transverse mass^3^ $m_{\mathrm {T}}> 40\mathrm {\ GeV}$, missing transverse momentum $E_{\mathrm {T}}^{\mathrm {miss}}>25\mathrm {\ GeV}$, and at most two jets (to reject $t\bar{t}$ backgrounds). The triggers are fully efficient for electrons and muons satisfying the offline $p_{\mathrm {T}}$ requirements.

In events in which two jets are reconstructed, only the jet with the highest $p_{\mathrm {T}}$ is studied.

#### $t\bar{t}$ sample

Top quark pair events in which exactly one of the $W$ bosons produced by the top quarks decays to an electron or a muon are selected as described in Ref. [48]. The event selection requires that exactly one electron or muon is reconstructed and that it matches the trigger accepting the event. Background suppression cuts of $m_{\mathrm {T}}>40\mathrm {\ GeV}$ ($m_{\mathrm {T}}>60\mathrm {\ GeV}$) and $E_{\mathrm {T}}^{\mathrm {miss}}>25\mathrm {\ GeV}$ ($E_{\mathrm {T}}^{\mathrm {miss}}>20\mathrm {\ GeV}$) in the electron (muon) channel, and at least four jets with $p_{\mathrm {T}}^\mathrm{\ jet} > 25\mathrm {\ GeV}$, $|$JVF$|>0.75$ and $|\eta ^\mathrm{jet} |<2.5$ are also required. Two of the selected jets must be identified as arising from a $b$-quark ($b$-tagged) using the MV1 algorithm, which combines several tracking variables into a multi-variate discriminant, with the 60 % efficiency working point [49].

After this selection, the background contamination in the $t\bar{t}$ sample is of the order of 10 % and consists mainly of events from $W/Z$+jets or single top-quark production. The contribution from multi-jet background after the requirement of two $b$-tagged jets is about $4\,\%$ [48]. The background contamination in the selected data sample has no sizable impact in the studies performed. The change in the results when including the background in the analysis is small, and the sample is therefore assumed to be pure $t\bar{t}$.

## Jet labelling in Monte Carlo simulation

One natural definition of the partonic flavour of a jet in a Monte Carlo event is given by matching the jet to the closest outgoing parton (in $\Delta R$) from the matrix-element calculation, which represents a fixed-order QCD event record. In generators with $2\rightarrow 2$ matrix elements, such a matching scheme is clear only for the two leading jets at most. To simplify the task for analyses using different MC simulations, jets are matched to the highest-energy parton in the parton shower record within a $\Delta R$ equal to the radius parameter of the jet algorithm. Using this method, only a small fraction of the jets (${<}1$ % around jet $p_{\mathrm {T}}=50$ $\mathrm {\ GeV}$ and fewer above 100 $\mathrm {\ GeV}$) are not assigned a partonic flavour. Studies with Pythia 6 and MadGraph indicate that jets with significant energy contributions from more than one distinct parton (e.g. overlap of initial- and final-state radiation) are rare in the samples used. The jet isolation requirement restricts the wide-angle QCD radiation of the jet and further guarantees the accuracy of the labelling based on the parton shower record.

Jets are identified as originating from $c$- and $b$-quarks by requiring one $c$- or $b$-hadron with $p_{\mathrm {T}}>5$ $\mathrm {\ GeV}$ in the MC record within a $\Delta R$ equal to the radius parameter of the jet. Jets with two $c$- or $b$-hadrons are identified as including a gluon splitting to $c\bar{c}$ or $b\bar{b}$. Both classes are considered separately from quark- and gluon-jets. The labelling of $b$-jets supersedes that of $c$-jets, which itself supersedes the quark and gluon labelling. In the samples used, other than $t\bar{t}$, the fraction of heavy-flavour jets is relatively small. The variables used for quark- and gluon-jet discrimination are sufficiently different for each of these jet types to require an independent treatment.

In MC event generators with matching schemes [25, 50, 51], it is possible to use the outgoing partons from the matrix-element calculation to label jets. Only jets above the matching scale can be identified in this manner, and only in exclusively showered events (i.e. events with the same number of jets at the matrix-element level and after showering). To avoid the need to tag jets originating from partons created in the parton shower, the matching scale must be chosen to be much lower than the minimum $p_{\mathrm {T}}$ of the jets for which the tagger is designed and commissioned. Labelling of jets based on the highest-energy parton is consistent with labelling based on the matrix-element calculation for isolated jets in the samples used here. The former is therefore used in this paper.

For the construction of templates and the examination of data, only ensembles of jets are considered. The parton record of the MC simulation is not used. Instead, the fractions of quark- and gluon-jets in each sample are calculated using the matrix-element event record, and only these fractions are used to describe the average composition of the jet ensemble.

## Determination of quark-jet and gluon-jet properties

In previous theoretical [5] and experimental [40] studies, the jet width and the number of tracks associated with the jet were found to be useful for identifying the partonic origin of a jet. As discussed in Sect. 1, the larger colour factor associated with a gluon results in the production of a larger number of particles and a softer hadron $p_{\mathrm {T}}$ spectrum after the shower. To define the optimal discriminant, several jet properties are examined for their ability to distinguish the partonic origin of a jet and for their stability against various experimental effects, including pile-up. As these jet properties depend on the jet kinematics, the analysis of the properties and the resulting discriminant are separated into bins of jet $p_{\mathrm {T}}$ and $\eta$. The $p_{\mathrm {T}}$ bin width is dictated by a combination of the jet resolution and the number of available events in data, and the $\eta$ bins coarsely follow the detector features.

### Discriminating variables

Useful discriminating variables, such as the number of particles associated with a jet, may be estimated using either the number of charged-particle tracks in the inner detector or using the number of topological clusters of energy inside the jet [40]. Although they are limited to charged particles, and thus miss almost half of the information in a typical jet, jet properties built from tracks have three practical advantages over calorimeter-based properties. First, they may include particles that have sufficiently low $p_{\mathrm {T}}$ that they are not measured by the calorimeter, or which are in the regime where the ID momentum measurement is more accurate than the energy measurement of the calorimeter. Second, charged particles bend in the magnetic field of the ID. Additional particles from the underlying event brought into the jet produce a background in the calorimeter, and particles that are sufficiently bent are lost to the calorimeter jet. However, both classes of particles can be correctly assigned using their momenta calculated at the interaction point. Third, tracks can be easily associated with a specific vertex. This association dramatically reduces the pile-up dependence of track-based observables. Similar arguments hold in the calculation of jet shape variables.

The variables surveyed as potential inputs to the quark/gluon tagging discriminant are:

- Number of reconstructed tracks ($n_\mathrm{trk}$) in the jet.
- Calorimeter width:
  $$\begin{aligned} w = \frac{ \sum _i p_{\mathrm{T},i} \times \Delta R(i,\mathrm{jet})}{ \sum _i p_{\mathrm{T},i} }, \end{aligned}$$
  where the sum runs over the calorimeter energy clusters that are part of the jet.
- Track width, defined similarly to the calorimeter width but with the sum running over associated tracks.
- Track-based energy–energy-correlation (EEC) angularity:
  $$\begin{aligned} \mathrm{ang_\mathrm{EEC}}=\frac{ \sum _i \sum _j p_{\mathrm{T},i} \times p_{\mathrm{T},j} \times (\Delta R(i,j))^\beta }{ (\sum _i p_{\mathrm{T},i})^2 }, \end{aligned}$$
  where the index $i$ runs over tracks associated with the jet, $j$ runs over tracks associated with the jet while $j>i$, and $\beta$ is a tunable parameter [52, 53].

**Fig. 1:**
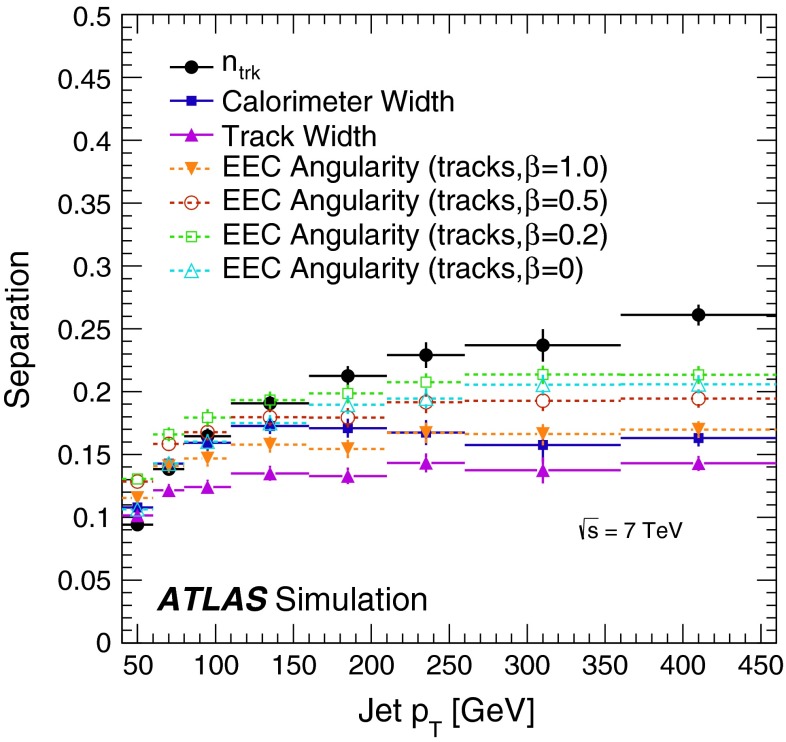
Separation power provided by different variables between quark- and gluon-jets as a function of jet $p_{\mathrm {T}}$ in the Pythia 6 dijet MC simulation for jets with $|\eta |<0.8$ built with the anti-$k_t$ algorithm with $R=0.4$

The discriminating power (“separation”) of a variable $x$ is calculated as in Ref. [54] to investigate the effectiveness of each variable in a quark/gluon tagger in a sample with equal fractions of quarks and gluons:

$$\begin{aligned} s=\frac{1}{2}\int \frac{(p_q(x)-p_g(x))^2}{p_q(x)+p_g(x)}\mathrm{d}x = \frac{1}{2}\sum _i\frac{(p_{q,i}-p_{g,i})^2}{p_{q,i}+p_{g,i}}, \end{aligned}$$

where $p_q(x)$ and $p_g(x)$ are the normalised distributions of the variables for quark- and gluon-jets, and where the second expression applies to histograms, with the sum running over the bins of the histogram. This definition corresponds to the square of the statistical uncertainty that one would get in a maximum-likelihood fit when fitting for the fraction of quark- or gluon-jets using the given variable, divided by the square of the uncertainty in the case of perfect separation. While this is not a variable that relates easily to quantities of interest for tagging, its interpretation is independent of the shape of the distributions, allowing for comparisons that are independent of the tagging efficiency. Using this definition, Fig. 1 shows, for different variables, the separation between quark-jets and gluon-jets as a function of jet $p_{\mathrm {T}}$ for jets built with the anti-$k_t$ algorithm with $R=0.4$ using the Pythia 6 dijet MC simulation. In this simulation, the two most powerful variables are the EEC angularity with $\beta =0.2$ and the number of tracks associated with the jet. The jet width built using the associated tracks is the weakest discriminant and the calorimeter-based width is somewhat stronger, and of comparable power to that of the EEC angularity with $\beta =1.0$.

Track-based variables show excellent stability against pile-up and significant discrimination power between quark- and gluon-jets. The dependence of the mean calorimeter width on the number of reconstructed vertices is about five times stronger than the dependence of any of the variables considered for the final discriminant and at low jet $p_{\mathrm {T}}$ is up to $\approx 1.5$ % per primary vertex. At high jet $p_{\mathrm {T}}$, the dependence is negligible for all variables. While it is possible to correct the inputs or to use a pile-up-dependent selection to allow the use of calorimeter-based variables without introducing a pile-up dependence in the tagger, such an approach is not pursued in this paper. Although Fig. 1 suggests using the charged particle multiplicity and the EEC angularity with $\beta =0.2$ to build the tagger, a larger linear correlation between these two variables makes this tagger perform worse at high $p_{\mathrm {T}}$ than the tagger built using the charged particle multiplicity and the track width. Furthermore, differences between data and MC simulation are reduced when using the latter tagger. For this reason, track width and $n_\mathrm{trk}$ are used to build the discriminant used in the rest of this paper. The linear correlations between $n_\mathrm{trk}$ and track width are at the 15 % level at low $p_{\mathrm {T}}$, increasing to 50 % at high $p_{\mathrm {T}}$. Thus, the variables add independent information about the properties of the jet. For EEC angularity with $\beta =0.2$, the linear correlation with $n_\mathrm{trk}$ is about 75 % with a weak dependence on $p_{\mathrm {T}}$. Still, the study of the EEC angularities and the evolution of their tagging performance as a function of $\beta$ is interesting for reasons discussed in Ref. [53]. Since this discussion is not relevant for the tagger developed in this paper, it is relegated to 1.

### Extraction of pure templates in data

To construct a discriminant, the properties of “pure” quark- and gluon-jets must be determined. As these properties depend on the modelling of non-perturbative effects, they are extracted from data to avoid reliance on MC simulations. The extraction can be performed using unbiased samples of pure quark- and gluon-jets or, alternatively, several mixed samples for which the admixture is well known theoretically. The use of pure samples is explored in detail in Sect. 6 as a validation procedure but is not used to determine the performance of the tagger in data, due to the limited number of events available and the difficulties in obtaining samples with negligible gluon and light-quark contaminations. The use of mixed samples is described below in detail, since it is used to create an operational tagger for data.

Distributions of properties of quark-jets or gluon-jets are extracted using the dijet and $\gamma +$jet event samples and the fraction of quark- and gluon-jets predicted by Pythia 6 with the AUET2B tune. For each bin $i$ of jet $\eta$, jet $p_{\mathrm {T}}$, and jet property (track width, number of tracks, or the two-dimensional distribution of the these), a set of linear equations is solved:

1 $$\begin{aligned} P_i(\eta ,p_{\mathrm {T}})&= f_{q}(\eta ,p_{\mathrm {T}}) \times P_{q,i}(\eta ,p_{\mathrm {T}}) \nonumber \\&\quad + f_{g}(\eta ,p_{\mathrm {T}}) \times P_{g,i}(\eta ,p_{\mathrm {T}}) \nonumber \\&\quad + f_{c}(\eta ,p_{\mathrm {T}}) \times P_{c,i}(\eta ,p_{\mathrm {T}}) \nonumber \\&\quad + f_{b}(\eta ,p_{\mathrm {T}}) \times P_{b,i}(\eta ,p_{\mathrm {T}}) , \end{aligned}$$

where $P_i$ is the value of the relevant distribution in bin $i$ of the distribution in the dijet or $\gamma +$jet sample, $f_{q}$ and $f_{g}$ are the light-quark and gluon fractions predicted by Pythia at a given $\eta$ and $p_{\mathrm {T}}$, and $P_{q,i}$ and $P_{g,i}$ are the values of the relevant distribution for quark- and gluon-jets in bin $i$ of the distribution. The fractions $f_{c}$ and $f_{b}$ for $c$-jets and $b$-jets are relatively small. They are taken from the MC simulation, together with the corresponding distributions $P_{c}$ and $P_{b}$. The same is true for the fractions and distributions for $g\rightarrow c\bar{c}$ and $g\rightarrow b\bar{b}$, not shown in Eq. 1 for brevity. By using the different fractions of light quarks and gluons in dijet and $\gamma +$jet events in each $p_{\mathrm {T}}$ and $\eta$ bin, the expected “pure” jet sample properties ($P_{q}$ and $P_{g}$) can be estimated. In these samples, the $b$-jet and $c$-jet fractions are typically below $5-10\,\%$. The studies are performed in three bins of $|\eta |$: $|\eta |<0.8$, $0.8<|\eta |<1.2$ and $1.2<|\eta |<2.1$.

An additional term $f_{\mathrm{fake}, i}(\eta ,p_{\mathrm {T}})\times P_{\mathrm{fake}, i}(\eta ,p_{\mathrm {T}})$ must be added to the distributions in the $\gamma +$jet sample to account for events in which the reconstructed (“fake”) photon arises from a jet with energy deposits mostly within the electromagnetic calorimeter. The term is estimated from data using a sideband counting technique, developed and implemented in Refs. [40, 44]. The method uses regions defined with varying levels of photon isolation and photon identification criteria, estimating the number of background events in the signal region from those in the background regions, after accounting for signal leakage into the background regions.

**Fig. 2:**
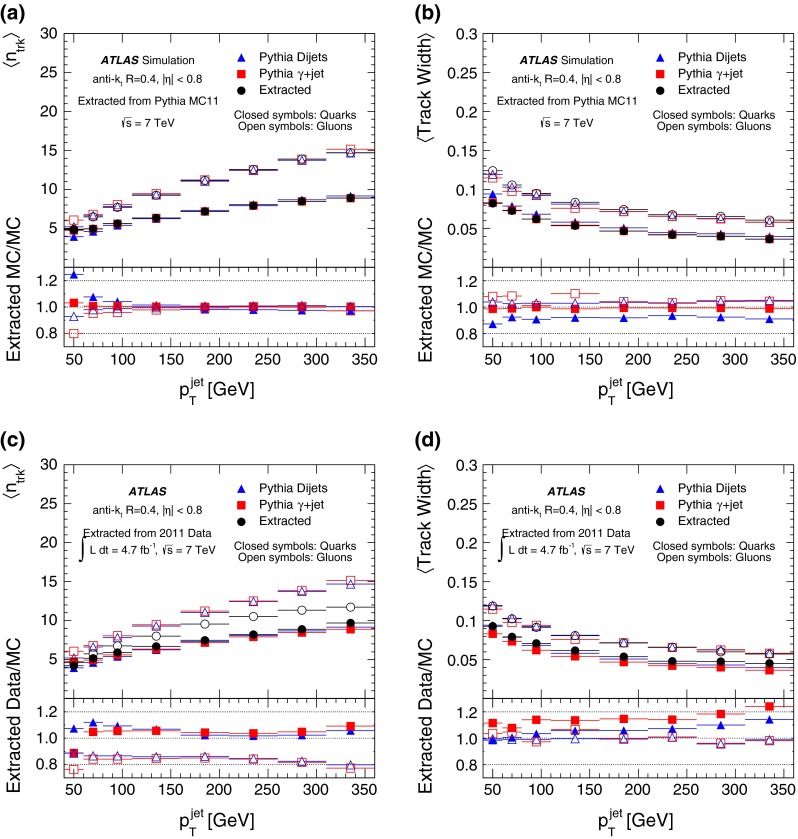
Average **a**, **c** $n_\mathrm{trk}$ and **b**,**d** track width for quark- (*solid symbols*) and gluon-jets (*open symbols*) as a function of reconstructed jet $p_{\mathrm {T}}$ for isolated jets with $|\eta |<0.8$. Results are shown for distributions obtained using the in-situ extraction method in Pythia 6 simulation (*black circles*, **a**, **b**)) or data (*black circles*, **c**, **d**), as well as for labelled jets in the dijet sample (*triangles*) and in the $\gamma \text {+jet}$ sample (*squares*). The *error bars* represent only statistical uncertainties. Isolated jets are reconstructed using the anti-$k_t$ jet algorithm with radius parameter $R=0.4$. The *bottom panels* show the ratio of the results obtained with the in-situ extraction method to the results in the dijet and $\gamma \text {+jet}$ MC samples

Knowledge of $P_x$ and $f_x$ for the dijet and $\gamma +$jet samples allows the extraction of pure quark- and gluon-jet $n_\mathrm{trk}$ and track width distributions from the data. The method can be tested in the MC simulation, comparing the properties of jets labelled in MC as quark- or gluon-jets and the properties extracted using Eq. (1) to demonstrate consistency. Figure 2 (top) shows the mean number of tracks and the mean track width as a function of the jet $p_{\mathrm {T}}$, separated using either the MC flavour labels or the extraction procedure in the same MC events for jets with $|\eta |<0.8$. Differences are observed between the average of the distributions in the dijet and $\gamma +$jet samples. This biases the extracted distributions for gluon-jets to be more like the gluon-jet properties in the dijet sample. The same is true for quark-jets and the $\gamma +$jet sample. The differences are larger at low $p_{\mathrm {T}}$ and for the track width distributions. The bias demonstrates a sample dependence, which is included as a systematic uncertainty on the performance of the discriminant built from these jet properties. These differences are, however, small compared to the differences between quark- and gluon-jets, demonstrating the sensitivity of the extraction method. Similar results are obtained for jets reconstructed with radius parameter $R=0.6$ and in other $|\eta |$ regions.

Figure 2 (bottom) shows the same MC simulation points as Fig. 2 (top), but here the data are used in the extraction. Relatively good agreement is found between data and Pythia AUET2B for the track width of gluon-jets and for the number of tracks in quark-jets. However, the mean number of associated tracks is significantly smaller for gluon-jets in the data than in the Pythia MC simulation. Similarly, the mean track width is larger in data than in the MC simulation for quark-jets.

Both these differences make gluon-jets and quark-jets more similar, reducing the discrimination power of these properties in data. Differences between the Pythia MC simulation and the data are also present in some of the other variables originally considered. These differences translate into non-negligible differences in the corresponding discriminants. For this reason, a fully data-driven tagger is built.

### Systematic uncertainties on the extraction procedure

The distributions extracted from data can be used to build a data-driven tagger, and to evaluate its performance in data. Uncertainties on the extracted pure quark- and gluon-jet property templates are thus propagated through as uncertainties on the performance of the tagger. The systematic effects considered can be classified into four categories: uncertainties on the input fractions ($f_{x,i}$), uncertainties on the input shapes ($P_{x,i}$), uncertainties on the fake photon background, and sample-dependence effects. This last category includes, for example, differences in quark-jet properties between samples, which result in different quark-jet rejection across the various samples. This effect is the one that causes the inconsistency in the extraction method, illustrated in Fig. 2. Sample-dependent effects are included as a systematic uncertainty rather than deriving a separate tagger for each event selection and MC simulation.

Because jets with different observable properties have different calorimeter response, an additional uncertainty in the jet energy scale arises from the modelling of the response as a function of the discriminant in the MC simulation. The resulting uncertainties on the jet energy response after tagging, in addition to the standard jet energy scale uncertainties, are determined to be below 1 % using a $\gamma \text {+jet}$ $p_{\mathrm {T}}$-balance study following the procedures described in Ref. [40].

#### Input fraction uncertainties

The fraction of quark- and gluon-jets can change when going from a leading-order calculation to a next-to-leading-order (NLO) calculation, changing the renormalisation/factorisation scale, or changing the PDF set.

The first two effects are examined by comparing the Pythia and MadGraph calculations, which have different renormalisation/factorisation scales and different ways of simulating real emissions. Similarly, the potential effect of the real emissions is also probed by comparing the matrix-element labelling and the highest-energy parton labelling. A 5 % uncertainty that is anti-correlated between quark- and gluon-jets is applied to cover the maximum variation seen in these comparisons. This uncertainty is uncorrelated amongst samples.

The potential mis-modelling of the fraction of quark- and gluon-jets in the MC simulation due to limitations of the PDFs is estimated using several PDF sets. The PDF sets use different fitting procedures (MRST, CTEQ and NNPDF sets), different orders in the perturbation theory expansion (MSTW2008lo for LO, CT10 for NLO) and different assumptions about the $$\alpha _s$$ calculation (MRST2007lomod for LO$$^*$$ and MRSTMCal for LO$$^{**}$$). A 5 % uncertainty, anti-correlated between quark- and gluon-jets, conservatively covers the differences between the various PDF sets. This uncertainty is considered uncorrelated between the dijet and $$\gamma \text {+jet} $$ samples because no significant trend is observed between the samples as the PDF set is changed.

#### Heavy-flavour input uncertainties

The fractions of $b$-jets and $c$-jets are varied by $\pm 20$ % in the dijet sample, following Ref. [55], and by $\pm 50$ % in the $\gamma +$jet sample to estimate a conservative uncertainty. As the fractions of $b$-jets and $c$-jets are small, these uncertainties remain sub-leading. The two input fractions are varied independently. The differences in the results obtained after the extraction of the pure quark- and gluon-jet properties are added in quadrature to obtain the total systematic uncertainty from this effect.

Uncertainties on the properties of $b$-jets are determined using a $t\bar{t}$ sample, described in Sect. 3. The purity of this sample is generally better than 95 %. An envelope 10 % uncertainty is included on the $b$-jet properties as a result of comparisons of $b$-jet properties between data and several MC simulations. The validation is performed using tagged jets. Differences between tagged and inclusive $b$-jets in the MC simulation are found to be within the assigned uncertainty.

For $c$-jets, several templates with 10 % increases in the rates of 2-prong, 3-prong, and 4-prong decays are used to estimate the effect of changes to the $c$-hadron decay. These different $c$-jet distributions are propagated through the extraction procedure and the largest difference is used as the systematic uncertainty on the performance of the tagger due to this effect.

#### Fake photon background uncertainty

Several variations in the background to the $\gamma +$jet sample are considered. The identification requirements used to define the regions for the background estimation method are changed, resulting in purity differences of up to 10 % for low-$p_{\mathrm {T}}$ jets. The same procedure is used to estimate an uncertainty on the jet properties in the fake background. An uncertainty of up to 4 % covers the changes in the means of the property distributions. These differences are propagated to the discriminant distribution to obtain a systematic uncertainty due to the purity estimate. An additional uncertainty covering the full shape correction to $P_\mathrm{fake}$ for signal leakage into the background regions of the sideband counting method is included as well, amounting to less than a 3 % change in the means of the property distributions.

#### Sample-dependence uncertainty

The application to a signal sample of a quark/gluon discriminant derived in a specific set of samples (or sample admixtures) rests upon the assumption that sample dependence is negligible, or that it can at least be parameterised as a function of visible properties of the event. One such property is the degree of isolation of the jet, which requires separate treatment. However, there are other effects, such as colour flow, that are much harder to constrain using the available data and may lead to a sample-dependence of jet properties.

Uncertainties on the jet properties are estimated first from differences between the $\gamma +$jet and dijet samples of the properties of quark- and gluon-jets. These are representative of the differences observed when comparing several different samples. Events generated with Pythia 6 and Herwig++ are also tested for this effect. The envelope of these variations is used to estimate a systematic uncertainty due to the sample dependence of the jet properties. This systematic uncertainty is sensitive to statistical uncertainties in the MC simulation. These statistical uncertainties are estimated and used to smooth the $p_{\mathrm {T}}$ dependence of the uncertainty following the procedure described in Ref. [56]. The sample dependence is consistently the dominant systematic uncertainty for all jet $p_{\mathrm {T}}$ bins. The differences between MC labelled samples derive from differences in observable properties in the dijet and $\gamma \text {+jet}$ samples. It is thus critical to consider these effects when estimating uncertainties on the tagging efficiency.

The properties of non-isolated jets differ from those of isolated jets, in general. In both the data and the MC simulation, isolated jet properties show no significant dependence on the $\Delta R$ to the nearest reconstructed jet for $\Delta R >0.7$. As the discriminant constructed here uses only jets satisfying this isolation criterion, no additional uncertainty due to the effect of jet non-isolation is applied.

An additional uncertainty arises from an incorrect description of the $p_{\mathrm {T}}$-dependence of the tagging variables for samples with a significantly different jet $p_{\mathrm {T}}$ spectrum from that of the dijet and $\gamma +$jet samples with which the discriminant was constructed. This accounts for the differences in bin-to-bin migrations in the various samples. As this uncertainty is dependent entirely on the sample to which the discriminant is applied, it is not explicitly included here.

## Validation with event-level kinematic cuts

The jet property templates extracted in the previous section can be further validated using high-purity quark- and gluon-jet samples. Largely following the work in Ref. [19], events are selected using basic kinematic cuts and event-level selection criteria to study purified samples of quark-jets and gluon-jets. These event selections are independent of the properties of individual jets and thus do not bias them. By including several different selections, the importance of colour flow and other sample-dependent effects can be evaluated using data.

The jets that are not tagged as $b$-jets in the $t\bar{t}$ sample, particularly in the case of events with exactly four jets, are mostly light-flavour jets. However, because of impurities introduced by gluon contamination and $W\rightarrow c\bar{s}$ decays, they are not sufficiently pure to be of use in this study.

### Validation of gluon-jet properties

As protons have a large gluon component at low $x$, inclusive low-$p_{\mathrm {T}}$ jet production at the LHC has a high rate of gluon-jet production. However, the fractions drop rapidly as jet $p_{\mathrm {T}}$ increases. Particularly at moderate- and high-$|\eta |$, the relative rate of gluon-jet production exceeds 50  % only below 150 $\mathrm {\ GeV}$ in jet $p_{\mathrm {T}}$.

Multi-jet events from QCD contain relatively more gluon radiation than the inclusive jet sample. The radiation is typically soft, implying that the third-leading jet will often be a gluon-jet. A useful kinematic discriminant that can further purify a multi-jet sample, discussed in Ref. [19], is:

2 $$\begin{aligned} \zeta =|\eta _3|-|\eta _1-\eta _2|, \end{aligned}$$

where $\eta _i$ is the pseudorapidity of the $i$th leading jet. A selection based on this variable can provide gluon-jet purity over 90 %, at the price of significantly reduced efficiency.

**Fig. 3:**
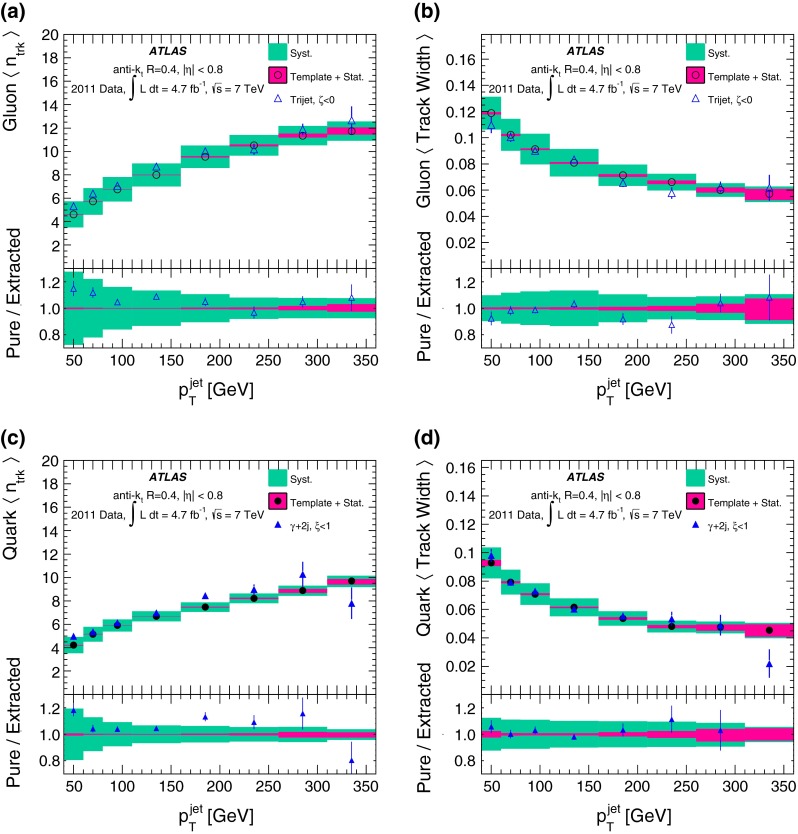
*Top*, the jet **a** $n_\text {trk}$ and **b** track width as a function of $p_{\mathrm {T}}$ for jets in a gluon-jet-enriched trijet sample (*triangles*) compared to gluon-jet extracted templates (*circles*) for $|\eta |<0.8$. *Bottom*, the jet **c** $n_\text {trk}$ and **d** track width as a function of $p_{\mathrm {T}}$ for jets in a quark-jet-enriched $\gamma$+jet sample (*triangles*) compared to quark-jet extracted templates (*circles*) for jets with $|\eta |<0.8$. Jets are reconstructed with the anti-$k_t$ algorithm with $R=0.4$. The *bottom panels* of the figures show the ratios of the results found in the enriched sample to the extracted results. *Error bars* on the points for the enriched sample correspond to statistical uncertainties. The *inner shaded band around the circles* and in the ratio represents statistical uncertainties on the extracted results, while the outer *error band* represents the combined systematic and statistical uncertainties

To evaluate the modelling of gluon-jet properties, events in data with $\zeta <0$ are compared to those extracted using the template technique described in Sect. 5. The track multiplicity and jet width are shown in Fig. 3a, b. The mean values of properties obtained using the purified and (regular) mixed samples generally agree within statistical and systematic uncertainties. Systematic uncertainties in this figure are calculated as detailed in Sect. 5.3, and symmetrised around the central value.

### Validation of quark-jet properties

Events containing photons are widely used as an enriched sample of quark-jets. By selecting events with photons produced in association with exactly one jet, a sample of quark-jets that is up to 80 % pure for jets with $p_{\mathrm {T}}>150$ $\mathrm {\ GeV}$ can be constructed. Although the further enrichment of quark-jets in this sample is difficult, it is possible to obtain higher purities using events with a photon and two jets [19]. If no other selection cuts are applied, these events have a lower quark-jet fraction than inclusive $\gamma$-jet production. However, a kinematic selection can help to identify jets seeded by the parton that is most likely to have radiated the photon. As that parton must have had electric charge, selecting these jets enhances the purity of quark-jets and rejects gluon-jets.

Following Ref. [19], a variable is defined that allows the kinematic separation of quark-jets and gluon-jets:

$$\begin{aligned} \xi =\eta _\text {jet~1}\times \eta _\gamma +\Delta R_{(\text {jet~2},\gamma )}, \end{aligned}$$

where $\eta _\gamma$ ($\eta _\text {jet~1}$) is the $\eta$ of the photon (leading jet), and $\Delta R_{(\text {jet~2},\gamma )}$ gives the difference in $\eta$–$\phi$ space between the sub-leading jet and the photon. By imposing a requirement on this variable, purities over 90 % can be achieved, although with a significant loss of events.

To evaluate the modelling of quark-jet properties, events with $\xi <1$ are compared in data with those extracted using the template technique described in Sect. 5. The track multiplicity and jet width are shown in Fig. 3c, d. The two sets of data agree within statistical and systematic uncertainties. These results also hold in higher $|\eta |$ bins and for jets reconstructed with the anti-$k_t$ algorithm with $R=0.6$.

Additionally, the production of a $W$ boson in association with a jet can be used to provide a relatively pure sample of quark-jets. A useful variable in constructing the sample is the jet “charge”, defined as

$$\begin{aligned} c_j = \frac{ \sum _i q_i \times \left| \varvec{p_i} \cdot \hat{j} \right| ^{1/2} }{ \sum _i \left| \varvec{p_i} \cdot \hat{j} \right| ^{1/2} } \end{aligned}$$

where the sums run over all tracks associated with the jet, $\hat{j}$ is a unit three-vector pointing in the direction of the jet momentum, $\varvec{p_i}$ is the track momentum three-vector, and $q_i$ is the track charge. This variable has been found to be useful in discriminating jets originating from positively charged quarks from those originating from negatively charged quarks [57–59]. The leading contribution to $W$ production results in a jet with charge opposite to that of the $W$ boson. The main backgrounds are from gluon-jets, including those in events with jets misidentified as leptons, which should have a charge distribution that is approximately Gaussian and centred at zero.^4^

A pure sample of $W$ events, selected as described in Sect. 3, is divided into events in which the leading jet has a charge with the same sign as the identified lepton (SS) and those in which the charge is opposite (OS). Templates are then constructed for jet properties in the SS and OS samples, and the SS sample is used to subtract the gluon-jet contribution from the OS template.

**Fig. 4:**
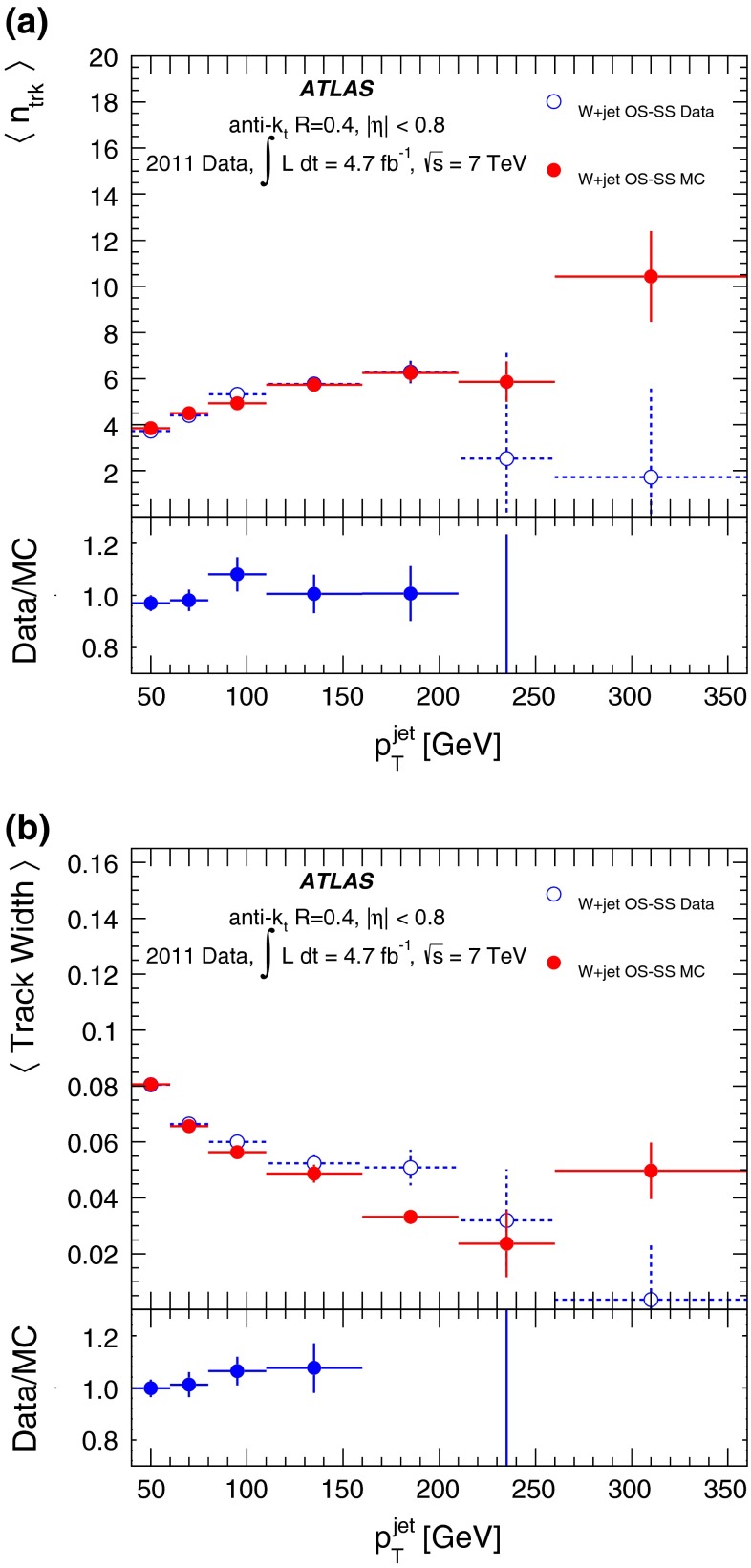
The jet **a** $n_\text {trk}$ and **b** track width as a function of $p_{\mathrm {T}}$ for quark-jets in an OS minus SS $W$+jet sample (see text) for $|\eta |<0.8$ in Pythia 6 MC simulation and in data. The *panels* show the ratio of the results in data to those in MC simulation

Comparisons between the mean of the OS minus SS distributions in data and MC simulation are shown in Fig. 4. The data show reasonable agreement with the MC simulation, generally within the statistical uncertainties. The points on these curves disagree at the 10 % level with extracted or purified quark-jet results shown in previous figures due to a non-closure effect in the method observed at low $p_{\mathrm {T}}$ in the MC simulation. Results from the $W+$1-jet MC simulation using generator-based labelling are in agreement with the quark-jet results from the dijet samples shown in Fig. 2.

## Light-quark/gluon tagger construction and performance

The discriminant for quark- and gluon-jets is based on a simple likelihood ratio that uses the two-dimensional extracted distributions of $n_\mathrm{trk}$ and track width for quark- and gluon-jets:

$$\begin{aligned} L=\frac{q}{q+g}, \end{aligned}$$

where $q$ ($g$) represents the normalised two-dimensional distribution for quark-jets (gluon-jets). A selection on $L$ is used in each bin to discriminate quark- and gluon-jets. This discriminant is built in bins of jet $p_{\mathrm {T}}$ and $\eta$. The two-dimensional distributions are first smoothed using a Gaussian kernel and then appropriately rebinned to build the discriminant distribution in such a way that all bins are populated sufficiently.

The performance of the tagger is determined using the two-dimensional extracted distributions of $n_\mathrm{trk}$ and track width in data and those obtained for labelled jets in MC simulations. Systematic uncertainties on the evaluated performance are estimated using alternative templates as described in Sect. 5.3. Table 1 summarises this performance for jets with $|\eta |<0.8$. The efficiencies for gluon-jets and quark-jets are evaluated only at certain operating points with fixed light-quark efficiency. Statistical uncertainties are evaluated using pseudoexperiments. Systematic uncertainties are combined in quadrature and affect both the quark- and gluon-jet efficiency in data. Large differences between MC simulation and data in the variables used translate into large scale factors in the gluon-jet efficiency. Practically, analyses using this tagger would apply the appropriate MC tagger to MC simulation and the data tagger to data. These scale factors are needed for each MC tagger to create event weights for the MC simulation, so that the efficiency in the MC simulation matches the measured efficiency in such analyses. Three representative $p_{\mathrm {T}}$ bins are shown in the table.

**Table 1:** Summary of the performance of the quark-jet tagger on quark- and gluon-jets in data and Pythia6 MC simulation for jets built with the anti-$k_t$ algorithm with $R=0.4$ and with $|\eta |<0.8$

	Monte Carlo		Data		Scale factor	
	$\epsilon _\mathrm{quark}$ (%)	$\epsilon _\mathrm{gluon} (\%)$	$\epsilon _\mathrm{quark} (\%)$	$\epsilon _\mathrm{gluon} (\%)$	SF$_\mathrm{quark}$	SF$_\mathrm{gluon}$
$p_{\mathrm {T}}=60$–$80 \mathrm {\ GeV}$	30	8.4	($30.0\pm 0.8^{+3.2}_{-5.3}$)	($11.9\pm 0.3^{+7.5}_{-2.9}$)	$1.00\pm 0.03^{+0.11}_{-0.18}$	$1.42\pm 0.04^{+0.89}_{-0.34}$
	50	21.0	($50.0^{+1.4+4.3}_{-1.3-6.8}$)	($26.6^{+0.8+7.1}_{-0.6-3.9}$)	$1.00^{+0.027+0.09}_{-0.026-0.14}$	$1.27^{+0.04+0.34}_{-0.03-0.19}$
	70	41.5	($70.0^{+1.7+3.9}_{-1.5-11.0}$)	($48.4^{+1.1+4.7}_{-0.9-6.0}$)	$1.00^{+0.024+0.06}_{-0.022-0.16}$	$1.17^{+0.03+0.11}_{-0.02-0.14}$
	90	69.9	($90.0^{+1.5+1.7}_{-1.3-3.3}$)	($80.2^{+1.0+5.6}_{-0.8-2.2}$)	$1.00^{+0.02+0.02}_{-0.01-0.04}$	$1.15^{+0.015+0.08}_{-0.012-0.03}$
$p_{\mathrm {T}}=110$–$160 \mathrm {\ GeV}$	30	5.7	($30.0\pm 0.6^{+2.8}_{-4.6}$)	($11.6^{+0.6+6.2}_{-0.4-4.6}$)	$1.00\pm 0.02^{+0.09}_{-0.15}$	$2.03^{+0.11+1.08}_{-0.08-0.81}$
	50	13.9	($50.0\pm 1.0^{+4.1}_{-6.1}$)	($24.3^{+1.2+7.4}_{-0.8-9.2}$)	$1.00\pm 0.02^{+0.08}_{-0.12}$	$1.75^{+0.09+0.53}_{-0.06-0.66}$
	70	29.7	($70.0^{+1.0+3.9}_{-1.1-8.5}$)	($45.3^{+1.5+4.6}_{-1.1-9.3}$)	$1.00^{+0.01+0.06}_{-0.02-0.12}$	$1.52^{+0.05+0.15}_{-0.04-0.31}$
	90	64.8	($90.0^{+0.5+2.0}_{-0.6-2.6}$)	($78.1^{+1.0+3.5}_{-0.6-6.0}$)	$1.00^{+0.006+0.02}_{-0.007-0.03}$	$1.21^{+0.02+0.05}_{-0.01-0.09}$
$p_{\mathrm {T}}=310$–$360 \mathrm {\ GeV}$	30	3.9	($30.0^{+5.0+2.1}_{-7.1-4.7}$)	($11^{+5+8}_{-7-4}$)	$1.00^{+0.17+0.07}_{-0.24-0.16}$	$2.8^{+1.4+2.0}_{-1.9-1.1}$
	50	10.3	($50.0^{+8.1+3.0}_{-11.6-8.3}$)	($23^{+10+8}_{-12-9}$)	$1.00^{+0.16+0.06}_{-0.23-0.17}$	$2.2^{+1.0+0.8}_{-1.1-0.9}$
	70	23.5	($70.0^{+7.2+3.1}_{-8.8-7.0}$)	($43^{+8+6}_{-12-10}$)	$1.00^{+0.10+0.04}_{-0.13-0.10}$	$1.81^{+0.35+0.23}_{-0.51-0.42}$
	90	58.9	($90.0^{+5.0+1.8}_{-4.9-3.1}$)	($80^{+6+4}_{-10-7}$)	$1.00^{+0.06+0.02}_{-0.05-0.03}$	$1.37^{+0.10+0.07}_{-0.17-0.11}$

The first error corresponds to the statistical uncertainty, while the second corresponds to the systematic uncertainty. The scale factor is the ratio of data to MC simulation

The difference in efficiency between data and MC simulation is particularly large for the tightest operating point at high $p_{\mathrm {T}}$. It improves for the loosest operating points and is generally better for the lowest $p_{\mathrm {T}}$ bins. The efficiencies extracted from data show a much weaker dependence on $p_{\mathrm {T}}$ than is suggested by Pythia 6. No strong dependence on $\eta$ is observed in any sample. The performance obtained here in Pythia 6 compares well with the generator-level studies presented in Ref. [5]. The systematic uncertainties are dominated by the uncertainty due to the sample dependence.

**Fig. 5:**
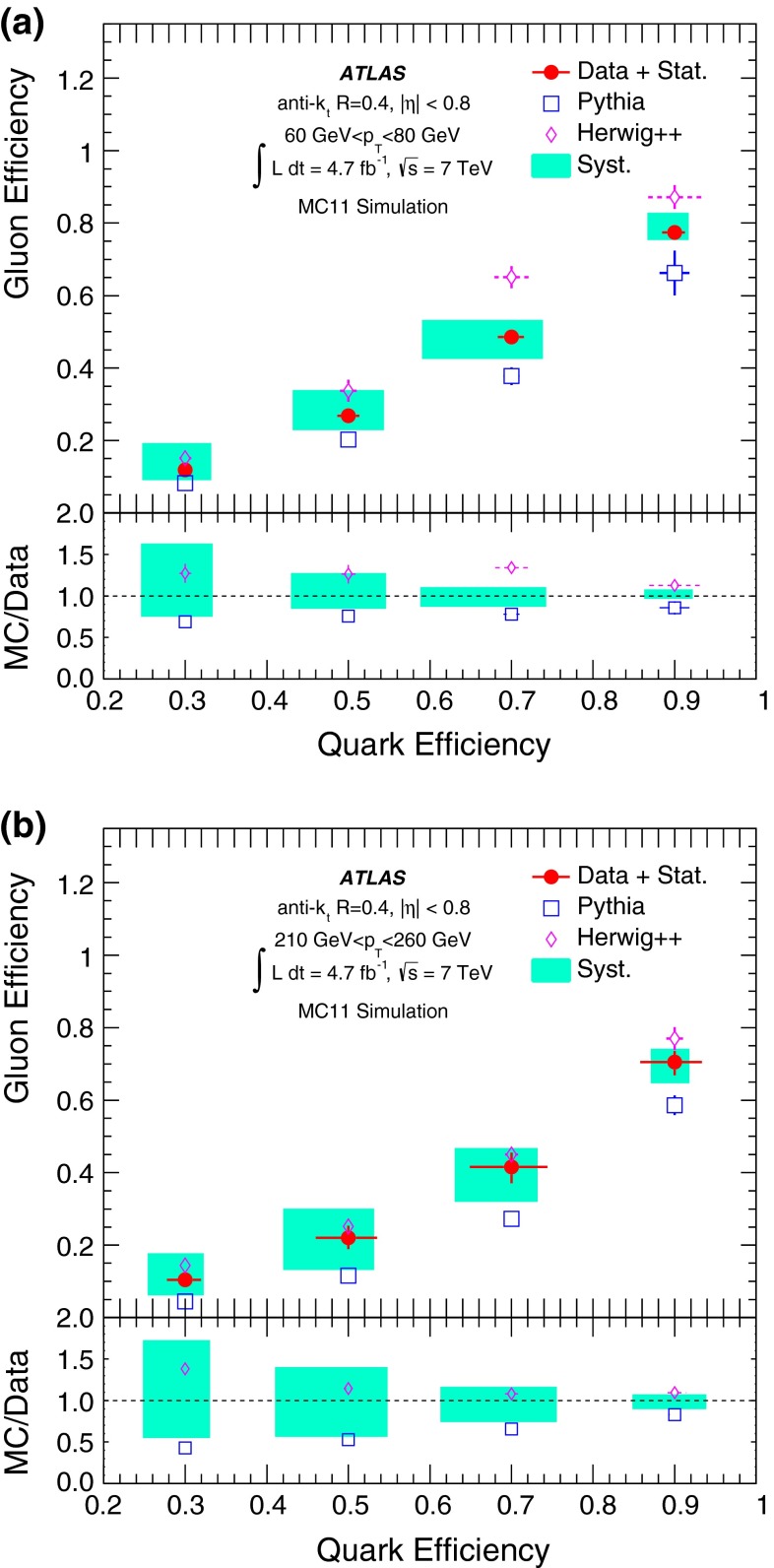
Gluon-jet efficiency as a function of quark-jet efficiency calculated using jet properties extracted from data (*solid symbols*) and from MC-labelled jets from the dijet Pythia 6 (*empty squares*) and Herwig++ (*empty diamonds*) samples. Jets with **a** $60<p_{\mathrm {T}}<80$ $\mathrm {\ GeV}$ and **b** $210<p_{\mathrm {T}}<260$ $\mathrm {\ GeV}$ and $|\eta |<0.8$ are reconstructed with the anti-$k_t$ algorithm with $R=0.4$. The *shaded band* shows the total systematic uncertainty on the data. The bottom of the plot shows the ratios of each MC simulation to the data. The *error bands* on the performance in the data are drawn around 1.0

The efficiencies of the tagger in MC simulation and in data are summarised in Fig. 5, where the performance estimated from labelled jets in dijet MC simulations and extracted data are shown. Two MC simulation-based taggers were used to produce this figure, one developed using distributions extracted in Pythia 6, which is applied to the Pythia 6 samples, and another derived from Herwig++, used for the Herwig++ samples. As expected from Sect. 5.2, the data do not agree well with either Pythia 6 or Herwig++. Differences between data and Pythia 6 are within systematic uncertainties at low $p_{\mathrm {T}}$, but are more significant at high $p_{\mathrm {T}}$ for those points for which a large sample is available in the data. The tagger performs worse in Herwig++ than on data at low $p_{\mathrm {T}}$ (Fig. 5a), but there is fair agreement in its performance for high $p_{\mathrm {T}}$ jets (Fig. 5b). Comparable results are observed for higher $|\eta |$ ranges, but with larger statistical uncertainties.

**Fig. 6:**
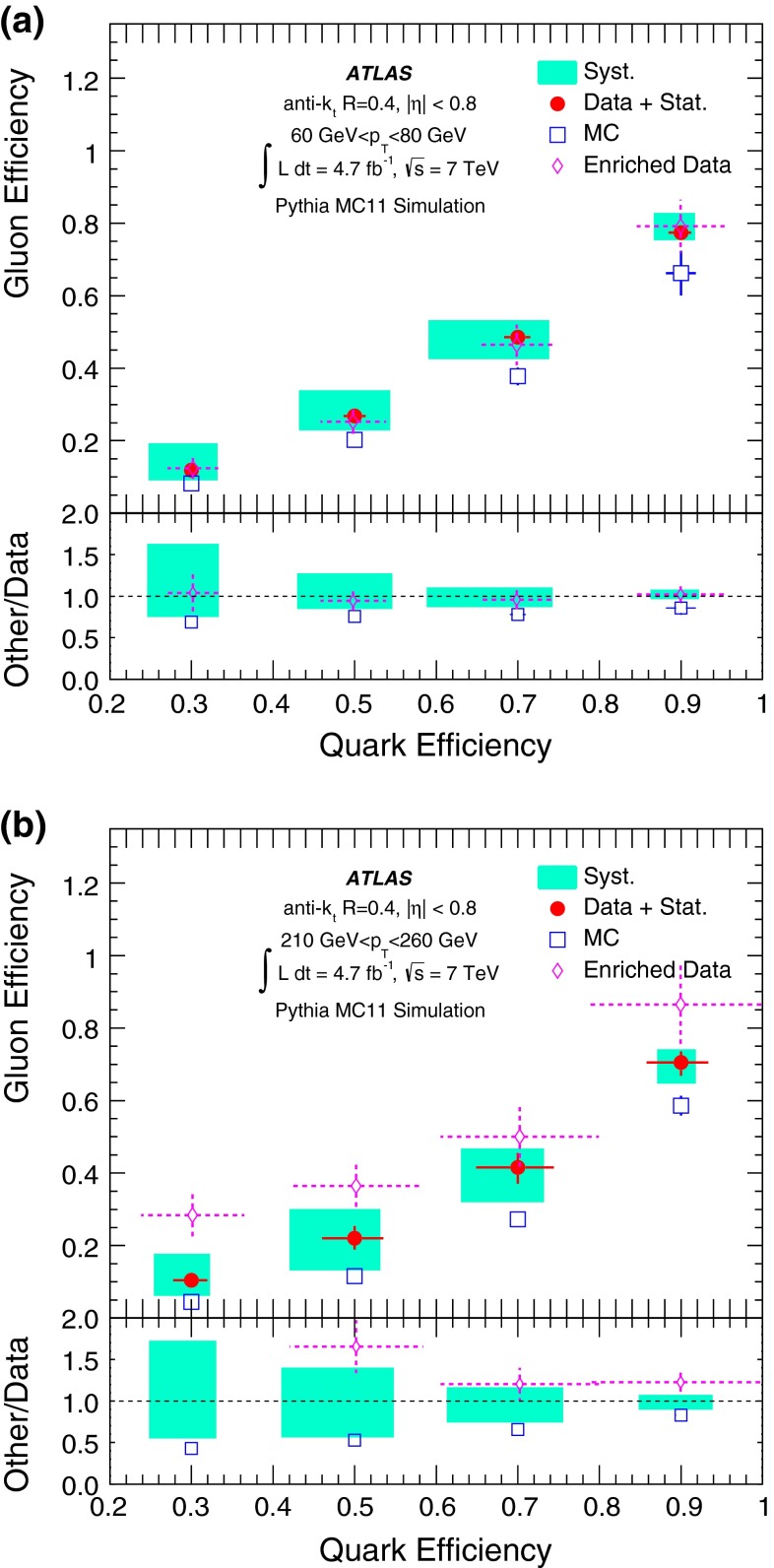
Gluon-jet efficiency as a function of quark-jet efficiency as calculated using jet properties extracted from data (*solid symbols*), purified in data through kinematic cuts (*empty diamonds*), and extracted from Pythia 6 MC simulation (*empty squares*). Jets with **a** $60<p_{\mathrm {T}}<80$ $\mathrm {\ GeV}$ and **b** $210<p_{\mathrm {T}}<260$ $\mathrm {\ GeV}$ and $|\eta |<0.8$ are reconstructed with the anti-$k_t$ algorithm with $R=0.4$. The *shaded band* shows the total systematic uncertainty on the data. The bottom of the plot shows the ratio of Pythia 6 MC simulation or the enriched data samples to the extracted data. The *error bands* on the performance in the data are drawn around 1.0

**Fig. 7:**
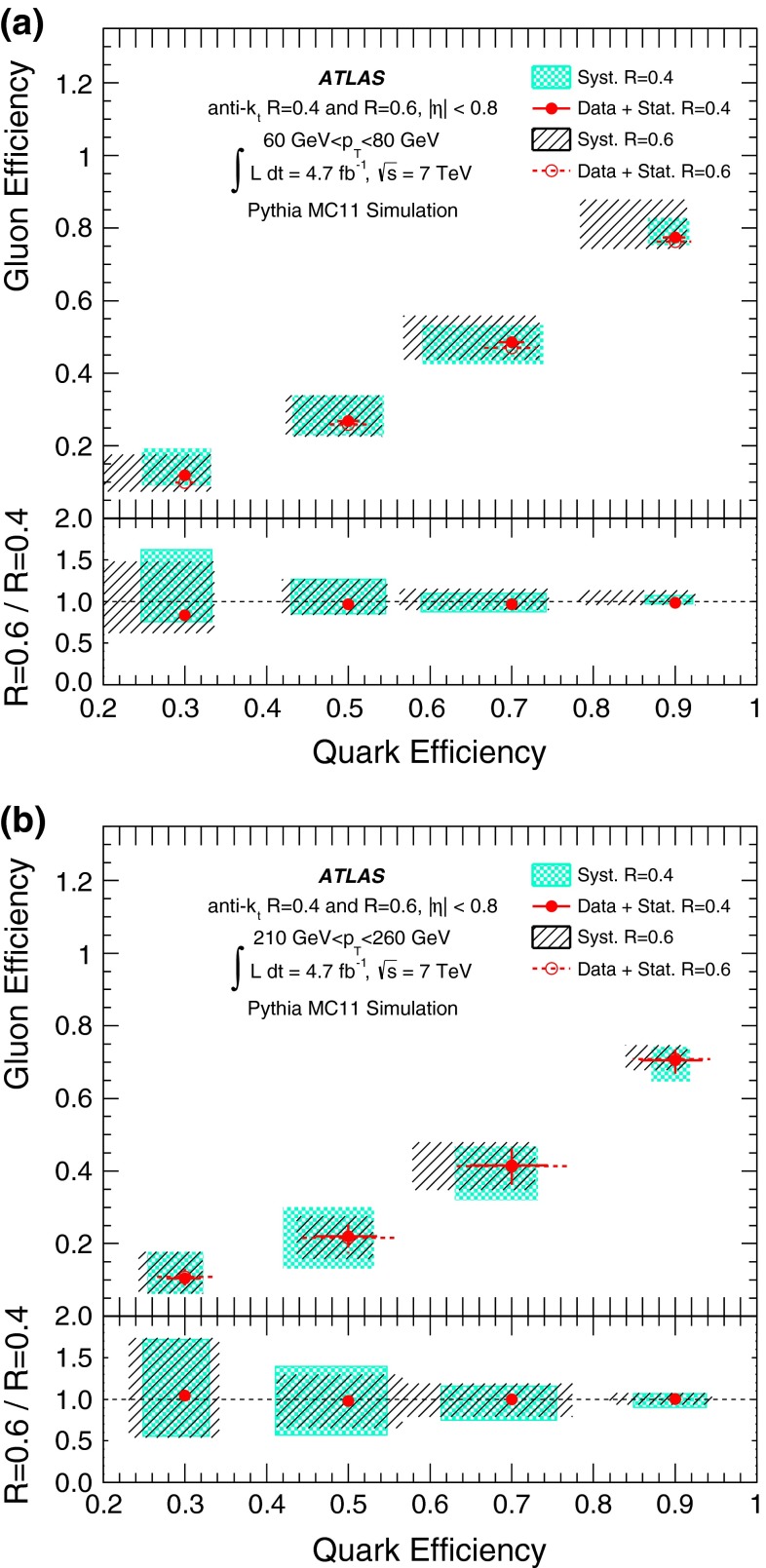
Gluon-jet efficiency as a function of quark-jet efficiency as calculated using extracted jet properties for jets with **a** $60<p_{\mathrm {T}}<80$ $\mathrm {\ GeV}$ and **b** $210<p_{\mathrm {T}}<260$ $\mathrm {\ GeV}$ and $|\eta |<0.8$ reconstructed with the anti-$k_t$ algorithm with $R=0.4$ (*solid symbols*) and $R=0.6$ (*empty symbols*). The *shaded* (*hashed*) *band* represents the total systematic uncertainty on the $R=0.4$ ($R=0.6$) data points. When hardly visible, the *empty symbols* are just behind the *solid symbols*. The bottom of the plot shows the ratio of the performance in data obtained for $R=0.6$ to that for $R=0.4$. *Error bands* are drawn around 1.0

The performance can also be calculated using the relatively pure samples obtained in trijet and $\gamma +$2-jet events (see Sect. 6). The efficiencies obtained using purified samples are compared in Fig. 6 to those obtained using the extracted discriminant distribution. The agreement within systematic uncertainties, particularly in Fig. 5a, further validates the extraction method. Some small differences, like those in Fig. 5b, should be expected from impurities in the quark and gluon purified samples. A comparison of performance in jets with radius parameters of $R=0.4$ and $R=0.6$ in data and MC simulation is shown in Fig. 7. The performance is comparable with the two jet sizes.

## Conclusions

Several variables that are sensitive to differences between quark- and gluon-jets were studied in various MC simulations and 4.7 fb$^{-1}$ of $\sqrt{s}=7$ $\mathrm {\ TeV}$ $pp$ collision data collected with the ATLAS detector at the LHC during the year 2011. Two of these variables, chosen to be relatively weakly correlated and stable against pile-up, were used to build a likelihood-based discriminant to select quark-jets and reject gluon-jets. Because of non-negligible differences in these variables between data and MC simulations, a data-driven technique was developed to extract the discriminant from the data and the MC simulations independently. This technique exploits significant, $p_{\mathrm {T}}$ dependent differences in the quark- and gluon-jet content between dijet and $\gamma +$jet samples.

A detailed study of the jet properties reveals that quark- and gluon-jets look more similar to each other in the data than in the Pythia 6 simulation and less similar than in the Herwig++ simulation. As a result, the ability of the tagger to reject gluons at a fixed quark efficiency is up to a factor of two better in Pythia 6 and up to 50 % worse in Herwig++ than in data. Gluon-jet efficiencies in data of $\approx 11\,\%$ and 80 % are achieved for quark-jet efficiencies of $\approx 30\,\%$ and 90 %, respectively. Relative uncertainties of $\approx 5{-}50\,\%$ ($\approx 3{-}20\,\%$) were evaluated for the estimate of these gluon-jet (quark-jet) efficiencies, with the uncertainties increasing for operating points with lower quark-jet efficiency. These uncertainties are dominated by differences in the properties of quark- and gluon-jets in the calibration samples (dijet and $\gamma +$jet) and are potentially caused by effects such as colour flow, which can make radiation around jets different for jets in different samples, even if they share the same partonic origin. These differences are predicted to be of different magnitude by the two generators studied and, without further insight, prevent final-state-dependent taggers to be developed. The differences between the properties in the two samples are typical of the variations of the properties observed in other samples studied.

The likelihood-based discriminants were studied independently in kinematically purified gluon-jet and quark-jet samples in data. Agreement is found within systematic uncertainty between the properties that are used to build the discriminant for the pure samples and the mixed samples. The same is true for the tagger efficiencies.

Because their properties differ, the same likelihood-ratio discriminant cannot be applied to non-isolated jets. However, using the methodology described in this paper, a discriminant for non-isolated jets with typical rejections and efficiencies comparable to those of the isolated-jet discriminant can be derived.

## Figures and Tables

**Fig. 8:**
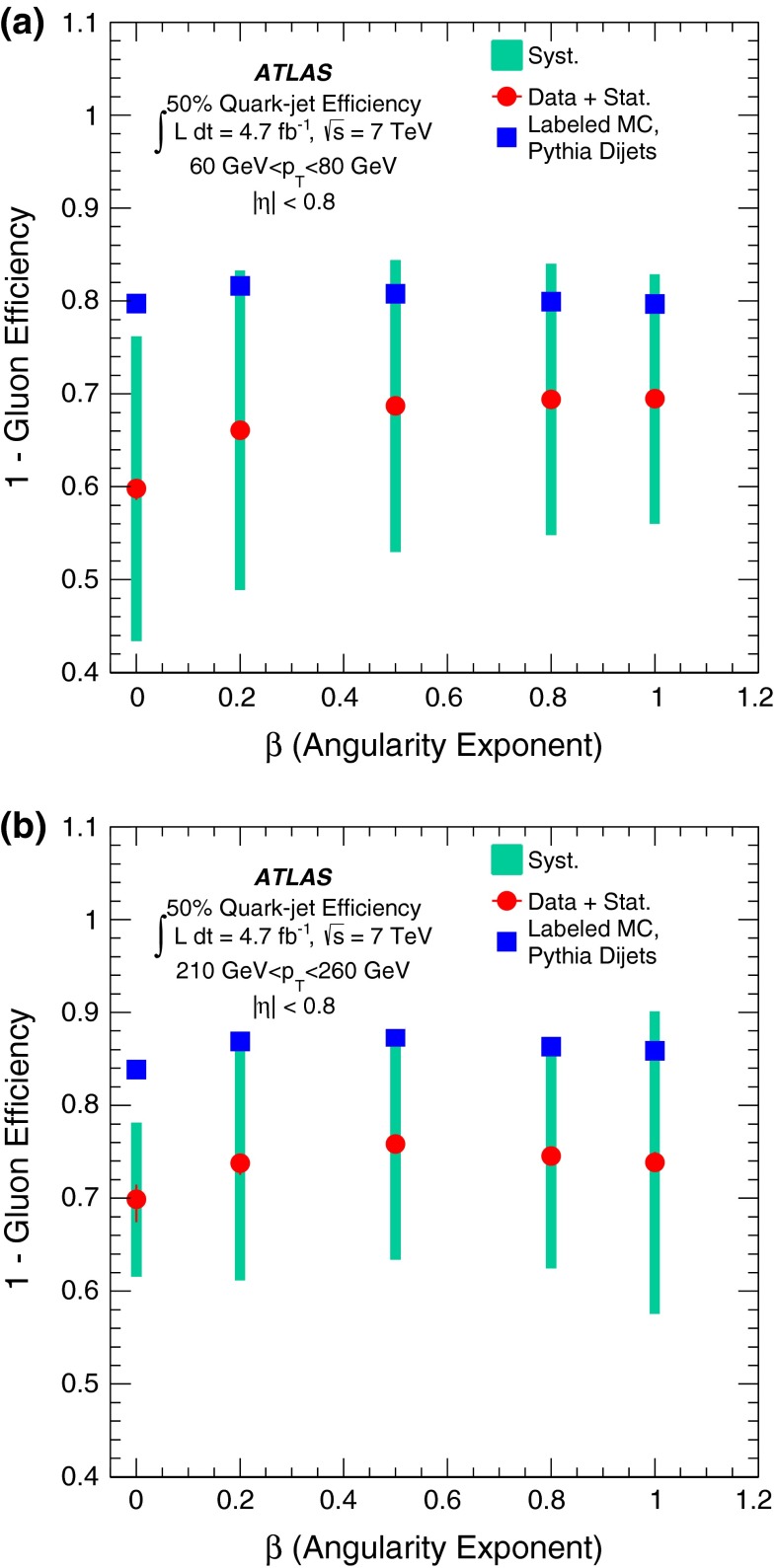
One minus the gluon-jet efficiency (for comparison with Ref. [53]) as a function of $\beta$ (see Sect. 5.1) for EEC angularities, calculated using extracted jet properties from data (*solid circles*) and dijet Pythia 6 MC simulation (*solid squares*) for jets with **a** $60<p_{\mathrm {T}}<80$ $\mathrm {\ GeV}$ and **b** $210<p_{\mathrm {T}}<260$ $\mathrm {\ GeV}$ and $|\eta |<0.8$ reconstructed with the anti-$k_t$ algorithm with $R=0.4$. The *shaded bands* represent the total systematic uncertainty on the data points

## Appendix A: Performance of EEC angularities

The EEC angularities described in Sect. 5.1 include a free parameter $\beta$ that affects the performance of the variables for quark/gluon discrimination. Recent studies [53] suggest that smaller values of the exponent $\beta$ provide stronger gluon-jet rejection for the same quark-jet efficiency. Figure 8 shows one minus the gluon-jet efficiency (for comparison with Ref. [53]) as a function of $\beta$, for a fixed quark-jet efficiency of 50 %, in data and Pythia 6 MC simulation. The MC simulation shows a weak dependence on $\beta$, with optimal performance for a $\beta$ value between 0.2 and 0.4. A similar trend is observable in data at high jet $p_{\mathrm {T}}$. At low jet $p_{\mathrm {T}}$, however, the performance falls off with lower $\beta$, with the highest few $\beta$ points showing comparable performance. The worst performance at all $p_{\mathrm {T}}$ is given by $\beta =0$, which uses the $p_{\mathrm {T}}$ of the tracks without angular information, emphasising the high-$p_{\mathrm {T}}$ tracks at the core of the jet for which the tracking momentum resolution is worse and inefficiencies and fakes due to shared hits between tracks in the detector become more common. At high jet $p_{\mathrm {T}}$, the point at $\beta =0$ shows reduced systematic uncertainties with respect to many of the other points, though the sample dependence is still significant.

It should be noted that the dependence on $\beta$ resembles more closely that found in Ref. [53] when using quark- and gluon-jets from either the dijet or $\gamma +$jet samples exclusively in building and testing the tagger in MC simulation. The template method developed in this paper is sensitive to the sample dependence of these variables. Since quark- and gluon-jets from the two samples used by the method show differences in these variables, the method is not capable of distinguishing this trend. The significant uncertainties on the data points in Fig. 8 are mostly from the uncertainties associated with this sample dependence. This serves to emphasise the importance of data-based validation of quark-jet/gluon-jet discriminants, as MC simulation may not correctly describe the jet properties observed in data, as well as the importance of correct MC event generator tunes that describe the jet properties and their potential sample dependence.

## Footnotes

1 ATLAS uses a right-handed coordinate system with its origin at the nominal interaction point (IP) in the centre of the detector and the $z$-axis along the beam pipe. The $x$-axis points from the IP to the centre of the LHC ring, and the $y$-axis points upward. Cylindrical coordinates $(r,\phi )$ are used in the transverse plane, $\phi$ being the azimuthal angle around the beam pipe. The pseudorapidity is defined in terms of the polar angle $\theta$ as $\eta =-\ln \tan (\theta /2)$, and the rapidity is defined as $\frac{1}{2}\ln {\left( \frac{E+p_Z}{E-p_Z}\right) }$, where $E$ is the object’s energy and $p_z$ is its momentum along the $z$-axis. The values of $\eta$, $\phi$, and $y$ are determined at the interaction vertex. The variable $\Delta R =\sqrt{(\Delta \eta )^2+(\Delta \phi )^2}$ is used to characterise the angular difference between two objects using their $\eta$ and $\phi$ directions.

2 A particle is considered stable and interacting if its lifetime is longer than 10 ps and it is neither a muon nor a neutrino.

3
$m_{\mathrm {T}}= \sqrt{E_{\mathrm {T}}^{\mathrm {miss}}\times E_{\mathrm {T}}^\ell \times (1-\cos (\Delta \phi ))}$, where $E_{\mathrm {T}}^{\mathrm {miss}}$ is the missing transverse momentum in the event, $E_{\mathrm {T}}^\ell$ is the lepton transverse energy (transverse momentum for a muon), and $\Delta \phi$ is the angle between the lepton and the $E_{\mathrm {T}}^{\mathrm {miss}}$ in the $\phi$ direction.

4 This is not quite the case, as the initial state at the LHC is more often positively charged than negatively charged.
